# *SETD8* inhibits ferroptosis in pancreatic cancer by inhibiting the expression of *RRAD*

**DOI:** 10.1186/s12935-023-02899-6

**Published:** 2023-03-18

**Authors:** Zekun Lu, Qiangsheng Hu, Yi Qin, Hao Yang, Bingkai Xiao, Weibo Chen, Shunrong Ji, Guangchen Zu, Zhiliang Wang, Guixiong Fan, Xiaowu Xu, Xuemin Chen

**Affiliations:** 1 Department of Hepatopancreatobiliary Surgery, the Third Affiliated Hospital of Soochow University, Changzhou, 213000 China; 2 Department of Thoracic Surgery, Shanghai Pulmonary Hospital, Tongji University School of Medicine, Shanghai, 200433 China; 3 Department of Pancreatic Surgery, Fudan University Shanghai Cancer Center, Shanghai, 200032 China

**Keywords:** *SETD8*, *RRAD*, Ferroptosis

## Abstract

**Background:**

As an oncogene, *SETD8* can promote tumour growth and tumour cell proliferation. This study aims to reveal the relationship between *SETD8* and ferroptosis in pancreatic cancer and its role in pancreatic cancer to provide a possible new direction for the comprehensive treatment of pancreatic cancer.

**Methods:**

The downstream targets were screened by RNA sequencing analysis. Western blot, Real-time Quantitative PCR (qPCR) and immunohistochemistry showed the relationship between genes. Cell proliferation analysis and cell metabolite analysis revealed the function of genes. Chromatin immunoprecipitation (CHIP) assays were used to study the molecular mechanism.

**Results:**

The potential downstream target of *SETD8*, *RRAD*, was screened by RNA sequencing analysis. A negative correlation between *SETD8* and *RRAD* was found by protein imprinting, Real-time Quantitative PCR (qPCR) and immunohistochemistry. Through cell proliferation analysis and cell metabolite analysis, it was found that *RRAD* can not only inhibit the proliferation of cancer cells but also improve the level of lipid peroxidation of cancer cells. At the same time, chromatin immunoprecipitation analysis (CHIP) was used to explore the molecular mechanism by which *SETD8* regulates *RRAD* expression. *SETD8* inhibited *RRAD* expression.

**Conclusions:**

*SETD8* interacts with the promoter region of *RRAD*, which epigenetically silences the expression of *RRAD* to reduce the level of lipid peroxidation in pancreatic cancer cells, thereby inhibiting ferroptosis in pancreatic cancer cells and resulting in poor prognosis of pancreatic cancer.

**Supplementary Information:**

The online version contains supplementary material available at 10.1186/s12935-023-02899-6.

## Background

Pancreatic cancer is a highly malignant tumour. It is the seventh leading cause of death among human cancers [1, 2]. With the development of medical technology, targeted drugs have been applied to breast cancer, lung cancer and other tumours and have achieved good clinical results [3, 4]. However, no effective molecular target for targeted therapy has been found for pancreatic cancer. Pancreatic cancer is resistant to most chemotherapeutic agents, so surgical treatment remains the only way to cure pancreatic cancer. However, due to its late discovery, most patients have lost best opportunity for operation by the time they see a doctor [5]. Therefore, it is particularly important to explore the molecular biological mechanism of the occurrence and development of pancreatic cancer. Understanding the molecular biological mechanisms of pancreatic cancer can provide new therapeutic targets and directions for the comprehensive treatment of pancreatic cancer. Although the overall 5-year survival rate of pancreatic cancer is less than 10%, in clinical practice and other authors' literature, a small proportion of pancreatic cancer patients have a relatively good prognosis [6].

Lysine methyltransferase *SETD8* (also known as *PR-SET7*, *SET8* or *KMT5A*) is a member of the SET domain family. Its important and most common function is to regulate the cell cycle and tumour growth [7, 8]; *SETD8* is unique among the KMTs discovered thus far. It is the only methyltransferase that can monomethylate histone H4 lysine 20 (*H4K20*). At the same time, the most important and common type of histone modification is methylation, and histone methylation is a common form of epigenetic disorder in tumorigenesis and development [9]. In addition, the methylation marker histone H4 lysine 20 (H4K20) is also considered to be an inhibitory signal of gene transcription, which plays a key role in DNA replication, DNA damage repair and silencing heterochromatin [10]. Therefore, enzymes that target the methylation of lysine residues in modified substrates are a potential direction of antitumour drug research. In our study, we found that SETD8 negatively regulates glucose metabolism and redox reactions in pancreatic cancer cells. Through gene expression profiling, we noticed that *SETD8* reduces the expression of *RRAD*, a closely related gene of glycometabolism (RAS associated with diabetes) [11]. Therefore, we speculate that *SETD8* may be involved in the regulation of lipid peroxidation in pancreatic cancer cells.

Ferroptosis is a new form of iron dependent programmed cell death that is different from apoptosis, necrosis and autophagy. It is characterized by lipid peroxidation of unsaturated fatty acids highly expressed on the cell membrane [12]. There is evidence that ferroptosis is associated with biological redox reactions and health. At the same time, inducing ferroptosis in cancer cells has great potential in cancer treatment. This is especially true in refractory malignant tumours that are resistant to traditional treatments such as radiotherapy and chemotherapy [13, 14]. Ferroptosis is closely related to RAS mutation of oncogenes. First, Brent R. Stockwell found that some small molecules, such as RAS-selective lethal 3 (RSL3) and erastin, can induce iron-dependent regulatory cell death different from other forms of cell death (such as apoptosis and necrosis) [15, 16]. Interestingly, nearly 95% of all pancreatic cancers have mutations in the KRAS gene [17]. However, research on pancreatic cancer and ferroptosis is insufficient. At the same time, we can analyse the level of lipid peroxidation in pancreatic cancer cells by *SETD8*. Therefore, we speculate that SETD8 may be involved in the regulation of ferroptosis in pancreatic cancer.

In this study, we studied the effect of methylation of histone H4 lysine 20 by lysine methyltransferase *SETD8* on ferroptosis in pancreatic cancer. We found that *SETD8* inhibited the occurrence of ferroptosis in pancreatic cancer. We proved that *RRAD* (RAS associated with diabetes) is a key target gene for *SETD8* and that *RRAD* can promote lipid peroxidation in pancreatic cancer cells. Mechanistically, *SETD8* inhibits the transcriptional activity of *RRAD* by binding to the promoter region of *RRAD*, thus downregulating the expression of *RRAD* and resulting in a decrease in the incidence of ferroptosis in pancreatic cancer cells. Therefore, high levels of *SETD8* and low levels of *RRAD* are closely related to poor prognosis in pancreatic cancer patients. At the same time, we found that the *SETD8*-*RRAD*-ferroptosis axis may be a potential target for the treatment of pancreatic cancer and provide a new strategy for the comprehensive treatment of pancreatic cancer.

## Materials and methods

### Cell culture

The human pancreatic cancer cell lines MIAPACA-2 and SW1990 were obtained from the American Type Culture Collection Center (ATCC, VA, USA) and cultured according to the ATCC-provided program. All cells used in the experiment were within 10 generations after thawing. All cell culture media contained 100 U/ml penicillin and 100 mg/ml streptomycin.

### Chemicals

The ferroptosis inducer RAS-selective lethal 3 (RSL3) and ferroptosis inhibitor ferrostatin-1 (Fer-1) were purchased from Selleckchem.

### Plasmids

The coding sequences of human *RRAD* and *SETD8* were cloned into the whole virus vector p pCDH-CMV-MCS-EF1-puro (SBI, USA) to produce the expression plasmids of *RRAD* and *SETD8*. To inhibit the expression of the target gene, the pLKO.1 TRC cloning vector (Addgene plasmid 10878, Watertown, MA, USA) was used. The 21 bp targets for *SETD8* were CCGAGGAACAGAAGATCAAAG and CGCAACAGAATCGCAAACTTA; the 21 bp targets for *RRAD* were CGTAGCTCGTAACAGCCGCAA and CACACCTATGATCGCTCCATT. The control interference shRNA (Addgene plasmid 1864) was used as a knockout control vector. The corresponding overexpression structures of *SETD8* and *RRAD* were obtained by using the pCDH-CMV-MCS-EF1-Puro vector (System Biosciences, Palo Alto, CA, USA), and empty body (EV) was used as a control. Lentiviruses are composed of the target gene expression vectors psPAX2 and pMD2. 293 T cells were added at a ratio of 4:3:1. Lentivirus particles were used to infect MIAPACA-2 and SW1990 cells, followed by puromycin screening to obtain stable cell lines.

### Western blot

Cells were first collected and washed twice with PBS, RIPA buffer mixed with protease and phosphatase inhibitors (Beyotime Biotechnology, Shanghai, China) was added, and the cells were lysed on ice for 30 min. Then, the protein concentration of the lysate was determined by a BCA protein analysis kit (Beyotime Biotechnology, Shanghai, China). Approximately 20 μg protein samples were separated on 10% SDS–polyacrylamide gels and then transferred to PVDF membranes (Millipore, Billerica, USA), and incubated with specific antibodies against SETD8 (Proteintech, 1:1000) and RRAD (Abcam, 1:1000). Next, the membrane was detected with a secondary antibody bound to HRP (protein, 1:5000). Finally, immunoblotting was incubated with an enhanced chemiluminescence detection kit (Millipore) and displayed by an imaging system (Clinx). The original figures of the western blots are all in Additional file 1.

### RNA extraction and real-time quantitative PCR

In summary, First, the cells were suspended in a 15 ml centrifuge tube and washed twice with PBS. Then, 2 × 10^7 cells were taken into a 1.5 ml EP tube and total RNA was extracted with Trizol reagent (Invitrogen, USA). cDNA was obtained by reverse transcription using the Takara primescript RT kit. Quantitative real-time PCR was used to determine the expression level of the target gene by an ABI 7900ht real-time PCR system (American Applied Biological Systems Company). β-Actin was used as the control, and the relative mRNA level was expressed by multiple changes compared with the control. The primers used were as follows: human *SETD8*: 5′-AAGATGTCCAAGCCCCGC-3′ (forward), 5′-TGTTCCTCGGACTTCATGGC-3′ (reverse); Human RRAD: 5′-ACATTTGGGAGCAGGACGG-3′ (forward), 5′-CTCTTGTTGCCCACGAGGAT-3′ (reverse); people β-Actin: 5′-CTACGTCGCCCTGGACTTCGAGC-3′ (forward), 5′-GATGGAGCCGCCGATCCACACGG-3′ (reverse). Subsequently, the delta-delta Ct method was used for data statistics. All tests were carried out in triplicate.

### Chromosome immunoprecipitation assay

Chromosome immunoprecipitation analysis was performed to evaluate the occupancy of *SETD8* on the *RRAD* promoter according to the instructions provided by the Magna CHIP A/G Chromatin Immunoprecipitation Kit (Darmstadt Millipore, Germany). A pair of primers was used to amplify the chromatin region of *RRAD*. The primer sequences were as follows: forward primer (5′–3′): AGTTGCTGCTTTTGGCTGATTGGGTT, reverse primer (5′–3′): AGTTGCTGCTTTTGGCTGATTGGGTT. Simply put, the cells were crosslinked with 1% formaldehyde for 10 min and then lysed and sonicated to an average size of 500 bp. The cross-linked protein/DNA complex was immunoprecipitated by anti-*SETD8* antibody (Santa Cruz Biotechnology, USA) and isotype control IgG (Cell Signaling Technology, 3900), incubated at 4 °C, bound to protein magnetic beads, eluted from the complex and purified for DNA. CHIP-ReCHIP was carried out basically the same as primary CHIP. The target DNA sequence was finally analysed on agarose gel for CHIP experiments.

### C11-BODIPY staining

Half a million cells were seeded into each well in a six-well plate (Corning) and then pretreated with a ferroptosis inducer for 24 h. Before flow cytometry, the cells were separated, resuspended and washed, and then stained with 2 μmol/l C11 BODIPY for 30 min. Then, the fluorescence intensity was detected by flow cytometry (Beckman).

### MDA and GSH/GSSG determination

The cells were placed in a 6-well cell culture plate (Corning). The treatment conditions were consistent with c11-bodipy analysis. After obtaining the cell homogenate, the protein concentration was measured using a BCA protein analysis kit (Beyotime), and then MDA was detected using the lipid peroxidation MDA Analysis Kit (Beyotime). After obtaining the MDA content, the ratio of MDA to protein concentration was calculated. The ratio of GSH/GSSG was measured according to the Beyotime GSH/GSSG assay kit and standardized using the protein concentration of the cell lysate.

### CCK-8 and colony formation test

We used the Cell Counting Kit-8 (Beyotime) for cell proliferation and toxicity experiments. The cells were inoculated into 96-well plates (1 per well) × 103 cells), and then 10 μl the Cell Counting Kit-8 (CCK-8) solution was added to each well at 0, 24, 48, 72, 96 and 120 h and incubated at 37 °C in 5% CO2 for 1.5 h, and then, the absorbance of each sample was measured at 450 nm wavelength using a microplate reader. In the cytotoxicity experiment, the cells were inoculated into 96-well plates (5000 cells per well) and then treated with a ferroptosis inducer and inhibitor. After 48 h, 10 μl CCK-8 solution was added to each well, incubated in 5% CO2 at 37 °C for 1 h, and finally, the absorbance of each sample was measured at 450 nm using a microplate reader. We tested cell viability according to the instructions of the Cell Counting Kit-8 reagent (Beyotime). For colony formation, pancreatic cancer cells (150 cells per pore) were inoculated in 6-well plates for 7–10 days. The colonies were fixed with 4% paraformaldehyde for 15 min, stained with 1% crystal violet for 30 min, and then counted.

### Tissue samples and immunohistochemical (IHC) staining

The clinical tissue samples used in this study were clinical PDAC tissue samples from 80 patients confirmed by surgery and pathology and approved by the ethics committee of the Affiliated Tumour Hospital of Fudan University (FUSCC). The immunohistochemical staining of paraffin-embedded tissues adopts a two-step scheme. First, The antigen was extracted by dewaxing hydration and antigen retrieval, and then the slide was incubated with the following primary antibodies: anti-*SETD8* (Proteintech) and anti-*RRAD* (Abcam). HRP binds affinity-purified sheep anti rabbit IgG (Proteintech) as a secondary antibody. Three different views were randomly selected under the microscope, and each slide was scored. According to the total area and intensity of staining, the protein expression level score was (1), < 5% of the total cells; (2), 5–25%; (3), 25–50%; (4), 50–75 and > 75%: (5). The final score was the average of the three views and was classified as follows: low (1 ≤ score < 3) and high (3 ≤ score ≥ 5).

### Statistical analysis

The experiment was repeated at least three times. All data were analysed by GraphPad Prism 8. Two-tailed unpaired Student’s t tests were used to compare the differences between the two groups. The χ^2^ test was used to analyse the relationship between the expression of SETD8 or RRAD and the corresponding clinicopathological features. Survival curves were drawn using the Kaplan‒Meier method and compared by the log-rank test. Differences were considered significant at *P < 0.05; **P < 0.01. *NS* means there was no significant difference.

## Results

### *SETD8* inhibits ferroptosis in pancreatic cancer cells

**Fig. 1:**
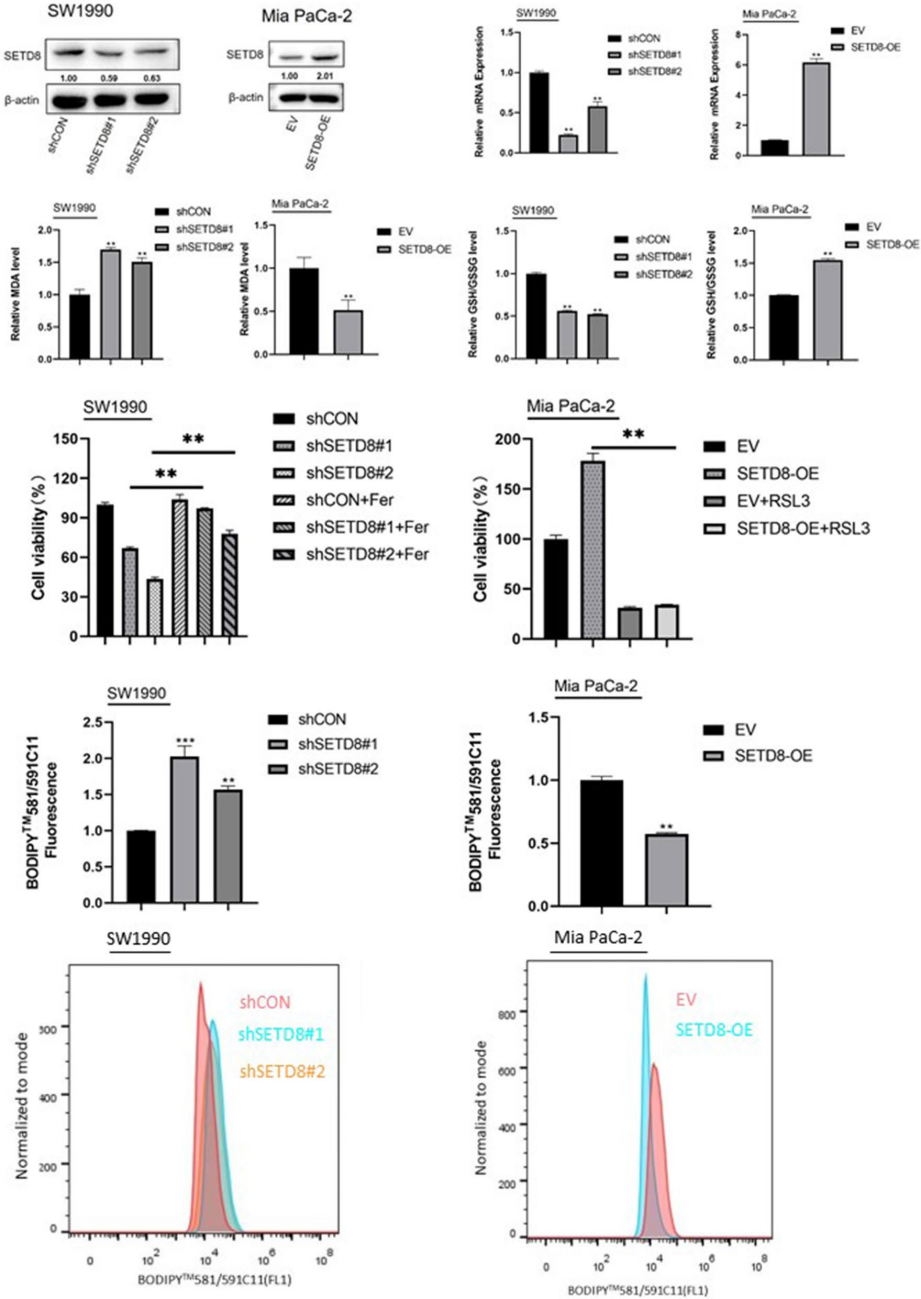
*SETD8* declines ferroptosis in pancreatic cancer cells. **A**, **B** The shRNAs against *SETD8* plasmid were transfected into SW1990 cell line. Plasmid overexpressing *SETD8* was transfected into Mia PaCa-2 cell line. Western blot analysis and qPCR analysis were performed to exam *SETD8* protein and mRNA levels, respectively. **C** The MDA was detected in *SETD8*-silenced SW1990 cell line and *SETD8*-overexpressed Mia PaCa-2 cell line. The results showed that *SETD8* could inhibit the expression of MDA. **D** The GSH/GSSG ratio was detected in *SETD8*-silenced SW1990 cell line and *SETD8*-overexpressed Mia PaCa-2 cell line. It was found that silencing *SETD8* could decrease the GSH/GSSG ratio and overexpression of *SETD8* could increase the GSH/GSSG ratio. **E** Cell viability was detected in the *SETD8*-silenced SW1990 cell line in the presence or absence of 2 μmol/l Fer and the *SETD*8-overexpressed Mia PaCa-2 cell line in the presence or absence of 2 μmol/l RSL3. The results showed that the cell viability decreased by *SETD8* knockdown could be reversed by the ferroptosis inhibitor Fer and the cell viability increased by *SETD8* overexpression could be reversed by the ferroptosis inducer RSL3. **F** Flow cytometry analyzed the fluorescence of BODIPY581/591C11 (lower) and the relative content was calculated (upper). The results showed that *SETD8* could reduce the level of intracellular lipid peroxidation. Two tailed unpaired Student t-test was used in the above experiments. *P < 0.05; **P < 0.01

The most common and important function of *SETD8* is to regulate the cell cycle and tumour growth. We silenced the expression of *SETD8* in the SW1990 cell line using two *SETD8*-specific shRNA expression lentiviruses (sh*SETD8*#1 and sh*SETD8*#2). At the same time, the expression of SETD8 was enhanced in the Mia PaCa-2 cell line. The efficiency of *SETD8* knockout and overexpression was confirmed by qPCR and Western blotting (Fig. 1a, b). We found that the level of lipid oxidation increased in the SW1990 cell line with *SETD8* silencing, but decreased with the Mia PaCa-2 cell line’s overexpression of *SETD8* (Fig. 1c). Therefore, we speculated that the expression level of glutathione as the main antioxidant [18] decreased in the cell lines with SETD8 knockout and increased in the Mia PaCa-2 cell lines with increased SETD8 expression. To confirm this hypothesis, we tested the GSH/GSSG ratio in the SW1990 cell line with silenced *SETD8* expression and the Mia PaCa-2 cell line overexpressing SETD8 (Fig. 1d). As expected, *SETD8* increased the expression of GSH. At the same time, GSH is closely related to GPX4, a key substance regulating ferroptosis [19]. Therefore, we used the BODIPY 581/591C11 probe to detect the level of lipid peroxidation in the SW1990 cell line with silenced *SETD8* expression and the Mia PaCa-2 cell line overexpressing *SETD8* (Fig. 1f). The results showed that *SETD8* inhibited the lipid peroxidation of cells. Lipid peroxidation is the most important marker of ferroptosis [20], and *SETD8* inhibits lipid peroxidation. Therefore, *SETD8* may inhibit ferroptosis. To verify this, we added the ferroptosis inhibitor ferrostatin-1 to the SW1990 cell line, which silenced *SETD8* expression. The ferroptosis inhibitor ferrostatin-1 (Fer) reversed the lipid peroxidation induced by *SETD8* silencing (Fig. 1e). A ferroptosis inducer (RSL3) was added to the Mia PaCa-2 cell line overexpressing *SETD8*. The results showed that a ferroptosis inducer (RSL3) could inhibit the increased cell viability of the cells overexpressing *SETD8* (Fig. 1e). This indicates that *SETD8* inhibits the occurrence of ferroptosis in pancreatic cancer cells.

### RNA expression profiling identified *RRAD* as a downstream target of *SETD8* regulating ferroptosis

**Fig. 2:**
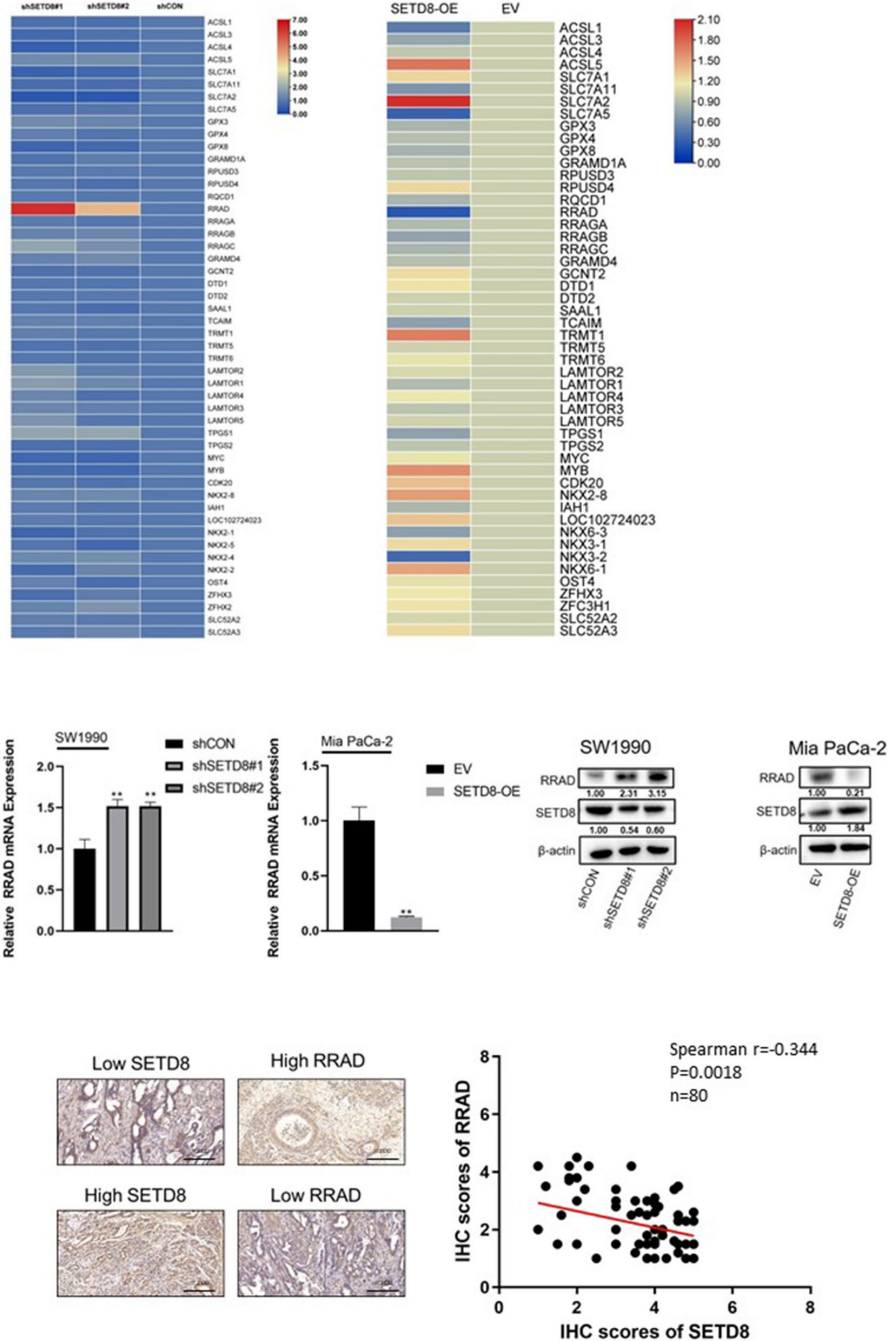
The downstream target gene of *SETD8* is *RRAD* and negatively regulates *RRAD* expression. **A** In the gene heatmap of knockdown of *SETD8* in SW1990 cells, *RRAD* was significantly overexpressed. In the heatmap of genes overexpressing *SETD8* in Mia PaCa-2 cells, *RRAD* was significantly decreased. These results indicated that *RRAD* was a downstream target gene of *SETD8* and was negatively regulated by *SETD8*. **B** qPCR analysis was performed to exam *RRAD* mRNA level. These results confirmed that *SETD8* knockdown led to upregulation of the *RRAD* expression level, while *SETD8* overexpression led to downregulation of the *RRAD* expression level. This further proves that *SETD8* negatively regulates *RRAD* expression. **C** Western blot was performed to exam *RRAD* protein level. The results showed that *SETD8* knockdown resulted in upregulation of the *RRAD* protein level, while *SETD8* overexpression led to downregulation of the *RRAD* protein level. This further indicates that *SETD8* negatively regulates *RRAD* expression. **D** Representative images of IHC staining of *SETD8* and *RRAD* in PDAC tumours and their correlation. *P* = 0.0018. It was further shown that SETD8 was negatively correlated with RRAD. *P < 0.05; **P < 0.01

To study the molecular mechanism by which *SETD8* regulates ferroptosis in pancreatic cancer cells, we examined the effect of *SETD8* knockout on gene expression profiles. Specifically, two different shRNAs targeting *SETD8* (sh*SETD8* # 1 and sh*SETD8* # 2) were used to silence the expression of *SETD8* in SW1990 cells, and *SETD8* was overexpressed in Mia PaCa-2 cells. After that, we compared the mRNA expression level differences between the above specific treatment cells by RNA sequencing. The results showed that a series of genes were upregulated and downregulated in SW1990 cells silenced by *SETD8* and Mia PaCa-2 cells overexpressed by *SETD8*. (Fig. 2a). The total number of genes in the final list is relatively limited, in part because two different sh*SETD8*s were transfected into the cell line to minimize the off-target effects and false-positive results of shRNAs. These include the RAS-related GTPase subfamily member *RRAD*. *RRAD* is also known as the RAS family diabetes-related gene, can inhibit the proliferation and migration of tumour cells, and has been identified as a tumour suppressor gene in many tumours [21–24]. It has been reported that epigenetic genes usually play a key role in cancer cells by inhibiting the expression of tumour suppressor genes [25]. Therefore, we confirmed the gene *RRAD* through qPCR and found that its expression was significantly upregulated with *SETD8* knockout, which was consistent with the sequencing data (Fig. 2b). At the same time, the expression of *RRAD* was also downregulated with *SETD8* overexpression (Fig. 2b). In addition, we further examined the effect of *SETD8* on *RRAD* at the protein level by Western blotting (Fig. 2c). Coincidentally, the result was consistent with that of qPCR. The expression of *RRAD* was significantly upregulated in *SETD8*-silenced cells and downregulated in *SETD8*-overexpressing cells. To further verify the relationship between *SETD8* and *RRAD* expression, IHC was performed in human PDAC tissue (Fig. 2d). The results showed that there was a negative correlation between the expression of *SETD8* and *RRAD* (r = − 0.344; *P* = 0.0018; n = 80).

### *RRAD* inhibits the proliferation of PDAC cell lines and is associated with a better prognosis in PDAC patients

**Fig. 3:**
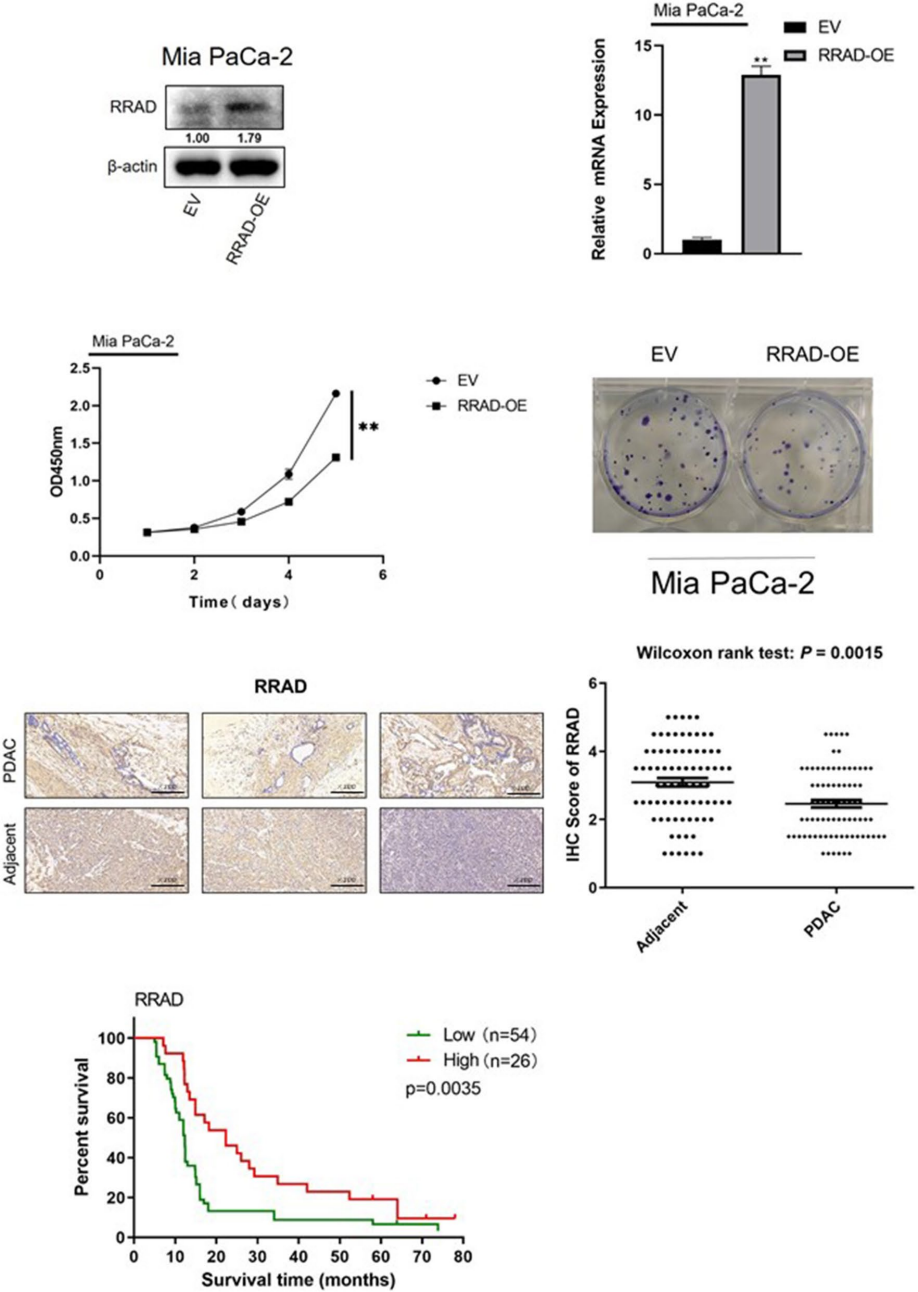
*RRAD* inhibits the proliferation of pancreatic cancer cells and is associated with the prognosis of patients with pancreatic cancer. **A**, **B** Western blot analysis and qPCR analysis were performed to exam *RRAD* protein and mRNA levels, respectively. The overexpression of *RRAD* was confirmed in Mia PaCa-2 cell line. **C** CCK-8 assay showed that overexpression of RRAD decreased the proliferation of pancreatic cancer cells. **D** Colony formation assay showed that overexpression of *RRAD* reduced the proliferation of pancreatic cancer cells. **E** Representative images of IHC staining of *RRAD* in PDAC tumour tissues and paired adjacent normal tissues. The results showed that *RRAD* was underexpressed in pancreatic cancer. **F** lHC analysis further confirmed that *RRAD* protein levels were lower in cancer tissues. wilcoxon rank test *P* = 0.0015. **G** Kaplan–Meier survival rate analysis for PDAC patients showed high *RRAD* expression was associated with longer over survival. *P < 0.05; **P < 0.01

To further study the function of *RRAD* in the development of pancreatic cancer, we overexpressed *RRAD* in the MIA PaCa-2 cell line and confirmed the efficiency of RRAD over expression by qPCR and Western blotting (Fig. 3a, b). By analysing the results of the following functional analysis, and we found that compared with the control group, the ability of *RRAD*-overexpressing cell lines to proliferate and form colonies decreased significantly (Fig. 3c, d), indicating that *RRAD* inhibited the proliferation of pancreatic cancer cells. At the same time, the expression of *RRAD* was closely related to the prolongation of overall survival (Fig. 3e, f, g). Overall, the above results indicate that the high expression of *RRAD* indicates that pancreatic cancer patients have a better prognosis and plays a role in inhibiting the occurrence and development of pancreatic cancer.

### *RRAD* promotes ferroptosis in pancreatic cancer

**Fig. 4:**
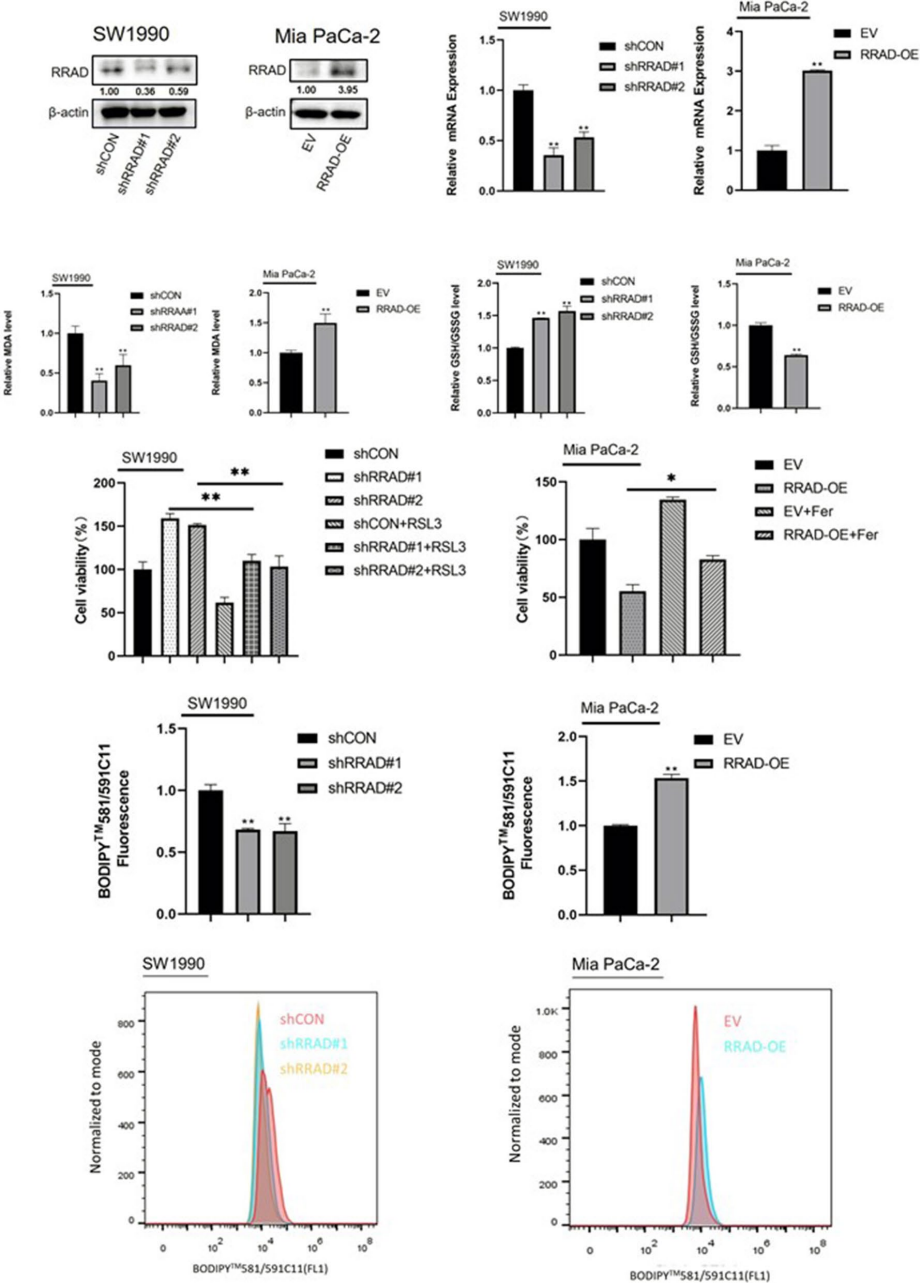
*RRAD* enhances ferroptosis in pancreatic cancer cells. **A**, **B** The shRNAs against *RRAD* plasmid were transfected into SW1990 cell line. Plasmid overexpressing *RRAD* was transfected into Mia PaCa-2 cell line. Western blot analysis and qPCR analysis were performed to exam *SETD8* protein and mRNA levels, respectively. **C** The MDA was detected in *RRAD*-silenced SW1990 cell line and *RRAD*-overexpressed Mia PaCa-2 cell line. The results showed that *RRAD* could promote the expression of MDA. **D** The GSH/GSSG ratio was detected in *RRAD*-silenced SW1990 cell line and *RRAD*-overexpressed Mia PaCa-2 cell line. It was found that silencing *RRAD* could increase the GSH/GSSG ratio and overexpression of *RRAD* could decrease the GSH/GSSG ratio. **E** Cell viability was detected in the *RRAD*-silenced SW1990 cell line in the presence or absence of 2 μmol/l RSL3 and the *RRAD*-overexpressed Mia PaCa-2 cell line in the presence or absence of 2 μmol/l Fer. The results showed that the cell viability increased by *RRAD* knockdown could be reversed by the ferroptosis inducer RSL3 and the cell viability decreased by *RRAD* overexpression could be reversed by the ferroptosis inhibitor Fer. **F** Flow cytometry analyzed the fluorescence of BODIPY581/591C11 (lower) and the relative content was calculated (upper). The results showed that *RRAD* could increase the level of intracellular lipid peroxidation. Two tailed unpaired Student t-test was used in the above experiments. *P < 0.05; **P < 0.01

*RRAD* can play a role in regulating the occurrence and development of pancreatic cancer. To further study its role in pancreatic cancer. We silenced the expression of *RRAD* in the SW1990 cell line by using two kinds of *RRAD*-specific shRNA expression lentiviruses (sh*RRAD*#1 and sh*RRAD*#2). At the same time, the expression of *RRAD* was enhanced in the Mia PaCa-2 cell line. The efficiency of *RRAD* knockout and overexpression was verified by qPCR and Western blotting (Fig. 4a, b). We found that the level of lipid oxidation decreased in the SW1990 cell line with silenced *RRAD* expression, while the level of lipid oxidation increased in the Mia PaCa-2 cell line overexpressing RRAD (Fig. 4c). Therefore, we speculate that the expression level of glutathione as the main antioxidant is increased in the cell line with knockout of *RRAD* expression and decreased in the Mia PaCa-2 cell line with increased *RRAD* expression. To test this hypothesis, we tested the GSH/GSSG ratio in the SW1990 cell line with silenced *RRAD* expression and the Mia PaCa-2 cell line overexpressing RRAD (Fig. 4d). As expected, *RRAD* decreased the expression of GSH. At the same time, GSH is closely related to GPx4 [19], a key substance regulating ferroptosis. Therefore, we used the BODIPY 581/591C11 probe to detect the level of lipid peroxidation in the SW1990 cell line, which silenced *RRAD* expression, and the Mia PaCa-2 cell line, which overexpressed *RRAD* (Fig. 4f). The results showed that *RRAD* promoted lipid peroxidation in pancreatic cancer cells. Lipid peroxidation is the most important marker of ferroptosis [20], and RRAD promotes lipid peroxidation. This suggests that *RRAD* may promote ferroptosis. To verify this, we added a ferroptosis inducer (RSL3) to the SW1990 cell line to silence *RRAD* expression. The ferroptosis inducer (RSL3) inhibited the increased cell viability of RRAD-silenced cells. The ferroptosis inhibitor ferrostatin-1 was added to the Mia PaCa-2 cell line to overexpress *RRAD*. The results showed that the ferroptosis inhibitor ferrostatin-1 partially reversed the lipid peroxidation caused by *RRAD* overexpression (Fig. 4e). This indicates that *RRAD* promotes the occurrence of ferroptosis in pancreatic cancer cells.

### *SETD8* suppresses ferroptosis in pancreatic cancer by downregulating* RRAD*

**Fig. 5:**
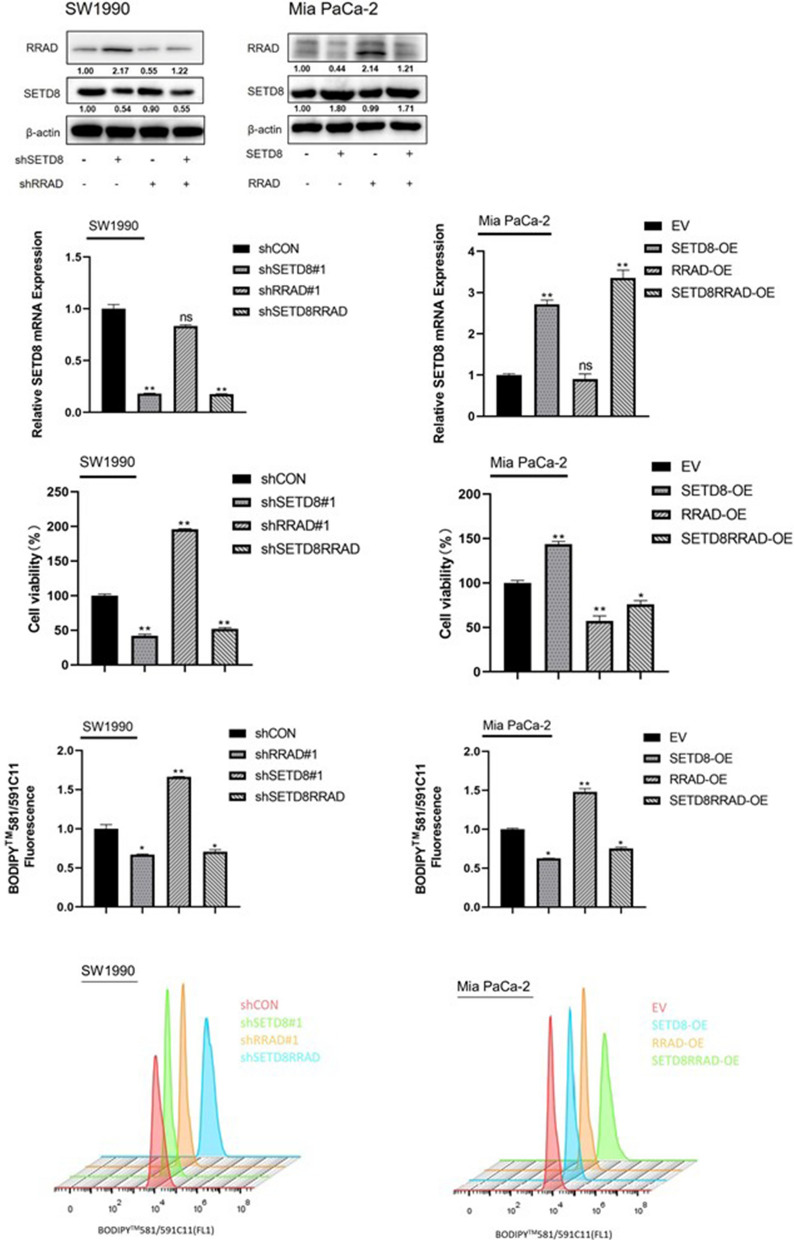
*SETD8* influences the occurrence of ferroptosis in pancreatic cancer through *RRAD*. **A**, **B** qPCR and Western blot assay confirmed the efficiency of shRNAs targeting *SETD8*, *RRAD* or both in the SW1990 cell line and the overexpression efficiency of *SETD8*, *RRAD* or both in the Mia PaCa-2 cell line. **C** Cell viability was detected in *SETD8*-, *RRAD*- or both-silenced SW1990 cell lines and in *SETD8*-, *RRAD*- or both-overexpressed Mia PaCa-2 cell line. The results showed that overexpression of *SETD8* enhanced cell viability, which was reversed by overexpression of *RRAD*. The increase in cancer cell activity caused by silencing *RRAD* expression was reversed by silencing *SETD8* expression. **D** Flow cytometry analyzed the fluorescence of BODIPY581/591C11 (lower) and the relative content was calculated (upper). The results showed that *SETD8* knockout induced elevated lipid peroxidation in cancer cells, which could be reversed by silencing *RRAD* expression. The elevated lipid peroxidation induced by *RRAD* overexpression was reversed by overexpression of *SETD8*. *P < 0.05; **P < 0.01

To further investigate how *SETD8* affects the occurrence of ferroptosis in pancreatic cancer, we speculated that *SETD8* may inhibit the occurrence of ferroptosis by inhibiting the expression of *RRAD*. Therefore, we constructed a stable pancreatic cancer cell line that silenced *SETD8* and *RRAD* and silenced *SETD8* and *RRAD* simultaneously in SW1990 cell lines. We also constructed stable pancreatic cancer cell lines that stably expressed *SETD8* and *RRAD* and overexpressed *SETD8* and *RRAD* in MIA PaCa-2 cell lines. Then, we performed qPCR and Western blotting to verify the efficiency of knockout and overexpression of the above cell lines (Fig. 5a, b). Then, we used the BODIPY 581/591c11 probe to detect the level of lipid peroxidation in the above treated cell lines at the same time (Fig. 5d). The results showed that the increase in lipid peroxidation levels in cancer cells caused by knockout of *SETD8* could be reversed by silencing the expression of the *RRAD* gene. The increase in lipid peroxidation caused by overexpression of *RRAD* can be reversed by overexpression of the *SETD8* gene.

To more directly observe the effect of the *SETD8* gene on the viability of pancreatic cancer cells, we detected the viability of these different pancreatic cancer cells. The results showed that overexpression of the *SETD8* gene increased the activity of cancer cells, which could be reversed by overexpression of the *RRAD* gene. The increase in cancer cell viability caused by silencing the expression of the *RRAD* gene can be reversed by silencing the expression of the *SETD8* gene (Fig. 5c). Therefore, the above results suggest that *SETD8* can inhibit ferroptosis in pancreatic cancer by downregulating the expression of *RRAD*.

### *SETD8* interacts with the promoter region of *RRAD* to inhibit its expression

**Fig. 6:**
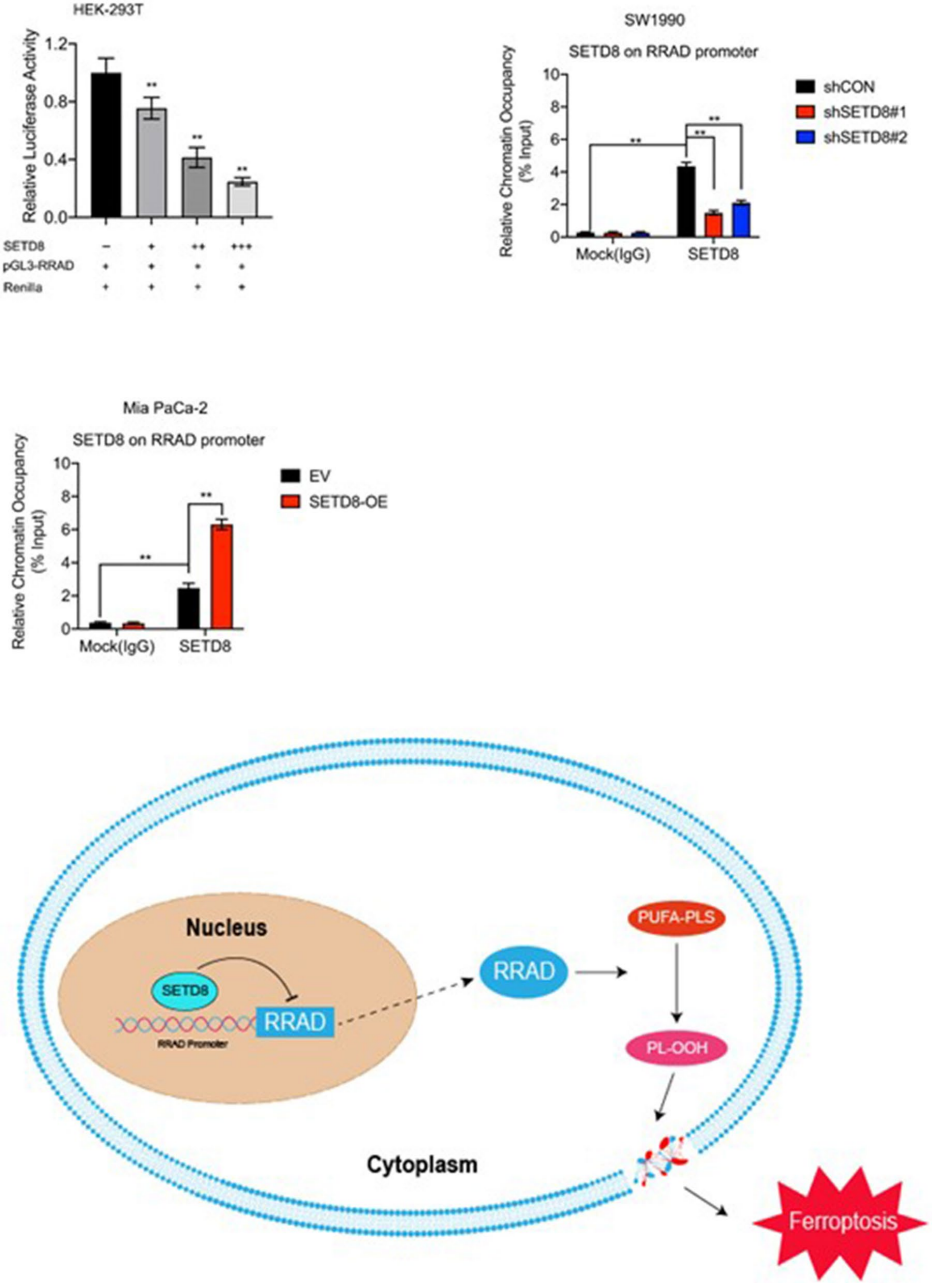
*SETD8* binds to the promoter of *RRAD* to inhibit its expression. **A** Dual-luciferase assay showed that the fluorescence intensity decreased gradually with the increase of *SETD8* expression. *SETD8* suppressed *RRAD* promoter activity in HEK293T cells. **B**, **C** CHIP‒qPCR analyses confirmed that *SETD8* specifically bound to the *RRAD* promoter in SW1990 cells and Mia PaCa-2 cells. **D** Schematic diagram of the mechanism of *SETD8* inhibiting cell ferroptosis. *SETD8* inhibits the transcription of *RRAD* by binding to the promoter of *RRAD*, which leads to a decrease in the level of lipid peroxidation, thus reducing the occurrence of ferroptosis in PDAC. Two tailed unpaired Student t-test was used in the above experiments. *P < 0.05; **P < 0.01

To better understand the mechanism by which *SETD8* affects *RRAD* expression, we carried out affinity purification and overexpression of *SETD8* labelled with FLAG (FLAG-*SETD8*) in Mia PaCa-2 cells. Cell extracts were prepared and affinity purified using an anti-flag affinity gel. Double luciferase reporter analysis was performed in HEK293T cells, and *SETD8* was overexpressed in the luciferase reporter driven by the *RRAD* promoter (Fig. 6a). The results showed that *RRAD* reporter activity decreased gradually with increasing *SETD8* overexpression. To further confirm the specific binding of *SETD8* to the *RRAD* promoter, SW1990 and MIA PaCa-2 cells were collected for chromatin immunoprecipitation (CHIP) analysis to verify the occupation of *SETD8* on the *RRAD* promoter. The results confirmed that *SETD8* occupied the *RRAD* promoter region (Fig. 6b, c). In general, *SETD8* inhibits *RRAD* transcription by interacting with the RRAD promoter, thus inhibiting ferroptosis in pancreatic cancer cells (Fig. 6d).

## Discussion

Pancreatic cancer is a highly malignant and aggressive tumour. The total survival rate of pancreatic cancer patients in the last 5 years is less than 10% [26, 27]. The RAS superfamily plays an important role in cell physiological activities. Its mutation or abnormal activation will promote the occurrence and progression of cancer [28, 29]. KRAS mutations can be observed in more than 95% of pancreatic cancers [17]. However, no obvious progress has been made in the treatment strategy of pancreatic cancer. Currently, the chemotherapy regimen is gemcitabine, anaxx, Forfield and albumin-bound paclitaxel. However, pancreatic cancer has strong chemoresistance, the prognosis of chemotherapy is still very poor [30]. Therefore, it is urgent to find compounds that can selectively kill RAS mutant cancer cells. Brent R. Stockwell found specific compounds that can kill RAS mutant cancer cells among tens of thousands of small compounds. In addition, they found that these compounds killed cancer cells in a manner different from apoptosis and necrosis [15, 16]; this method of death is named ferroptosis. Increasing evidence shows that ferroptosis has broad application prospects in the clinic. Ferroptosis holds great promise in cancer therapy, especially in treating tumors that have developed resistance to traditional therapies [31]. There has been evidence that drug-resistant cancer cells easily undergo ferroptosis. Therefore, ferroptosis can be used as a targeted therapy for cancer [14, 32]. At the same time, using nanomaterials as drug carriers to induce ferroptosis in cancer cells also provides another option [33]. In addition, studies have shown that the induction of ferroptosis in pancreatic cancer cells can enhance their sensitivity to chemotherapy, such as gemcitabine and cisplatin [34, 35]. Therefore, further study of the role of ferroptosis in pancreatic cancer will help to provide a new direction for the treatment of pancreatic cancer.

In this study, we found that lysine methyltransferase *SETD8*, a member of the SET domain family, plays an important and common role in regulating the cell cycle and tumour growth. It can increase the GSH/GSSG ratio in pancreatic cancer and reduce the level of lipid peroxidation. A high GSH/GSSG ratio and low lipid peroxidation can inhibit ferroptosis [36, 37]. We further demonstrated that *SETD8* can inhibit the occurrence of ferroptosis in pancreatic cancer. To elucidate the underlying mechanisms, we screened gene expression profiles. In *SETD8* downregulated genes, we found that RRAD, a member of the RAS-related GTPase subfamily and also known as the RAS family diabetes-related gene, can inhibit tumour cell proliferation and migration and has been identified as a tumour suppressor gene in many tumours [21–24]. We further found that *RRAD* could inhibit the proliferation of pancreatic cancer. In addition, *RRAD* can also reduce the GSH/GSSG ratio in pancreatic cancer and increase the level of lipid peroxidation. This indicates that *RRAD* can promote the occurrence of ferroptosis in pancreatic cancer. Our further experiments show that *SETD8* inhibits *RRAD* transcription and that the *SETD8* knockout-induced increase in lipid peroxidation levels in pancreatic cancer cells can be reversed by silencing *RRAD* gene expression. The increase in lipid peroxidation caused by overexpression of *RRAD* can be reversed by overexpression of the *SETD8* gene. Accordingly, ferroptosis inhibitors can save the cell viability reduced by low expression of *SETD8*. At the same time, based on the results of IHC analysis, the higher the level of *SETD8* or the lower the *RRAD* level, the worse the prognosis of pancreatic cancer patients. In order to further clarify how *SETD8* inhibits *RRAD* transcription. Through CHIP experiments, we demonstrated that *SETD8* regulates *RRAD* expression by specific binding to the *RRAD* promoter region.

*SETD8* has been shown to affect the progression of diabetic nephropathy by regulating *bach1* transcription [38]. It has also been found that *SETD8* can promote tumour cell growth and metastasis through the receptor tyrosine kinase *ROR1* [39]. Meanwhile, *SETD8* promoted the development of endometrial cancer by inhibiting the function of tumour suppressor genes through *H4K20* methylation and *p53* expression [40]. In this study, we demonstrated that *SETD8* can also promote tumor cell growth by inhibiting ferroptosis. Studies have found that *RRAD* can inhibit tumour cell proliferation, migration and Warburg effect by downregulating *ACTG1* expression [41, 42]. We demonstrate for the first time an association of *RRAD* with ferroptosis. *RRAD* can inhibit tumour cell growth through ferroptosis. The expression of *RRAD* was closely related to the prognosis of pancreatic cancer patients. Pancreatic cancer patients with high *RRAD* expression have a better prognosis than those with low *RRAD* expression. Furthermore, we found that *SETD8* inhibited the ferroptosis in pancreatic cancer by binding to the promoter region of *RRAD*, revealing the role of SETD8-RRAD-ferroptosis axis in the regulation of pancreatic cancer.

However, the limitation of this study is that it does not explain how *SETD8* combines with the promoter region of *RRAD* to regulate the transcription of *RRAD*. We speculate that *SETD8* may inhibit the transcription of *RRAD* by binding a transcription factor to the promoter of *RRAD*. *GPx4* is a key gene in the regulation of ferroptosis and we did not further investigate the relationship between *RRAD* and *GPx4*. At the same time, the role of *SETD8*-*RRAD*-ferrodeath axis in pancreatic cancer was not further verified in animal experiments.

Taken together, *SETD8* promotes the growth of pancreatic cancer cells by inhibiting ferroptosis. *RRAD* inhibits ferroptosis in pancreatic cancer cells. Meanwhile, low expression of *RRAD* is closely related to poor prognosis of pancreatic cancer patients. *STED8* inhibits the transcription of *RRAD* by binding to the promoter region of *RRAD* and thus reduces the expression of *RRAD*. Finally, it inhibits the ferroptosis of pancreatic cancer cells and promotes the proliferation of pancreatic cancer. These results reveal that the *SETD8*-*RRAD*-ferroptosis axis may provide potential therapeutic targets and predictors for the treatment of pancreatic cancer.

## Conclusions

Our study reveals the role of *SETD8*-*RRAD* in the occurrence of ferroptosis in pancreatic cancer. The combination of *SETD8* and the *RRAD* promoter subregion results in the inhibition of *RRAD* transcription, thereby affecting the occurrence of erroptosis in pancreatic cancer. These results may provide new strategies for the induction of ferroptosis in pancreatic cancer and provide a new direction for the comprehensive treatment of pancreatic cancer.

## Supplementary Information

**Additional file 1.** The original figures of the western blot.

## Data Availability

The datasets used and/or analysed during the current study are available from the corresponding author on reasonable request.

## Abbreviations

CHIP: Chromatin immunoprecipitation assay
H4K20: Monomethylate histone H4 lysine 20
Fer: Ferrostatin-1
RSL3: RAS-selective lethal 3
CCK-8: Cell counting kit-8
IHC: Immunohistochemistry
shRNAs: Short hairpin RNAs
