# Supplementary figures and images for: SETD8 inhibits ferroptosis in pancreatic cancer by inhibiting the expression of RRAD
Source: Cancer Cell Int. 2023 Mar 18;23:50. doi: 10.1186/s12935-023-02899-6 (PMC10024404; doi:10.1186/s12935-023-02899-6)

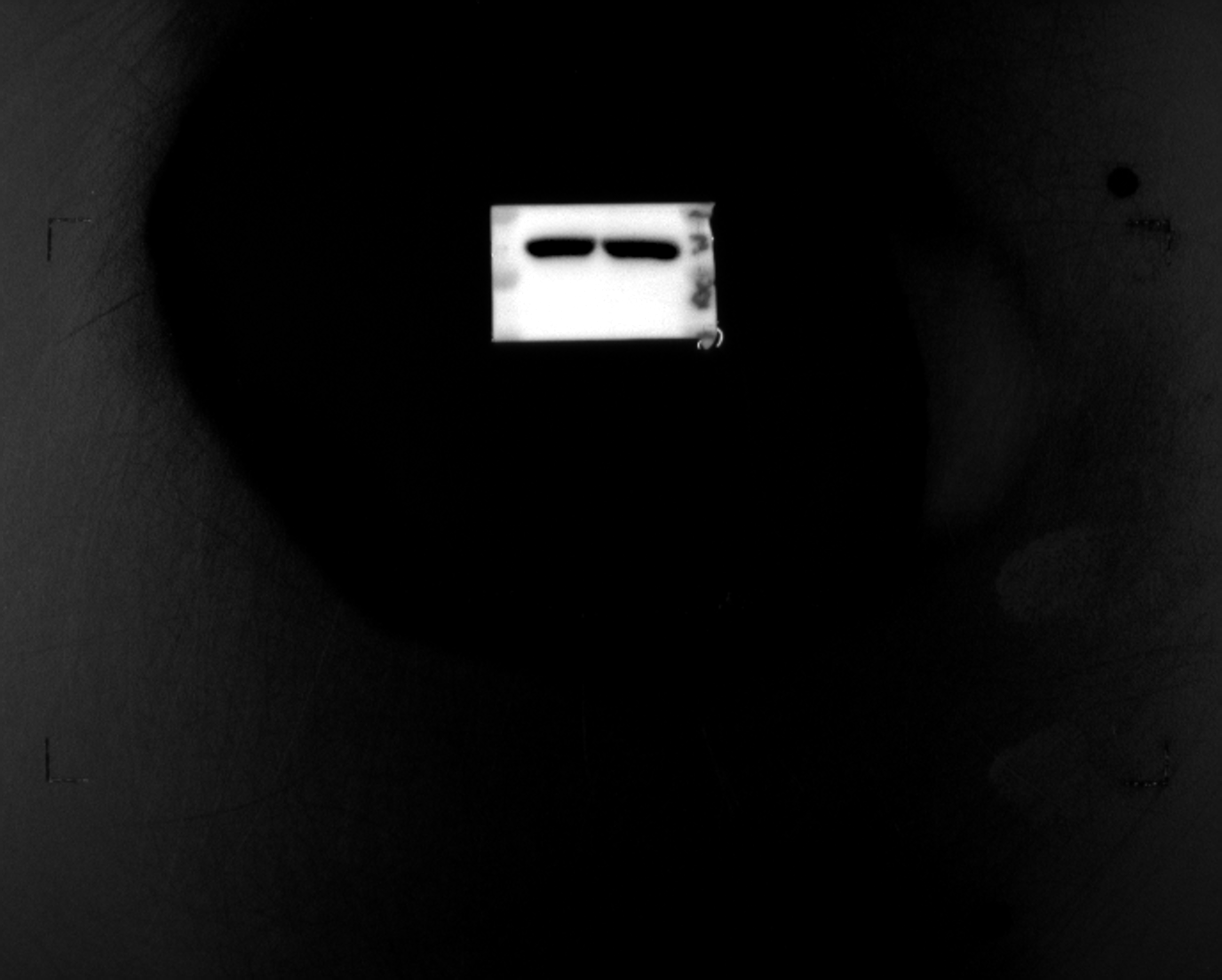

Supplement: Supplementary file 1 — Additional file 1. The original figures of the western blot. [file 12935_2023_2899_MOESM1_ESM.zip › Supplementary/Fig1 actin Mia PaCa-2.Tif]

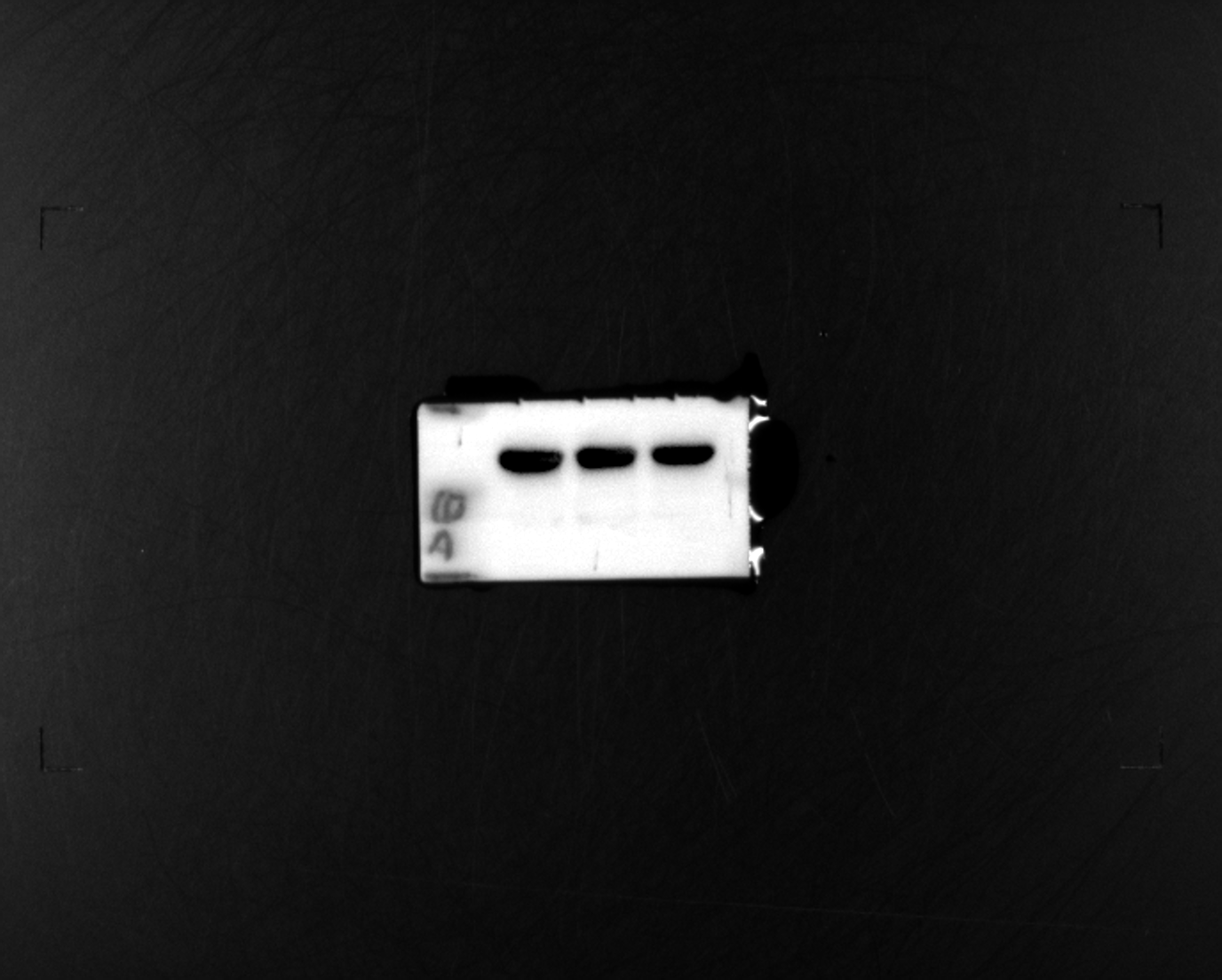

Supplement: Supplementary file 1 — Additional file 1. The original figures of the western blot. [file 12935_2023_2899_MOESM1_ESM.zip › Supplementary/Fig1 actin SW1990.Tif]

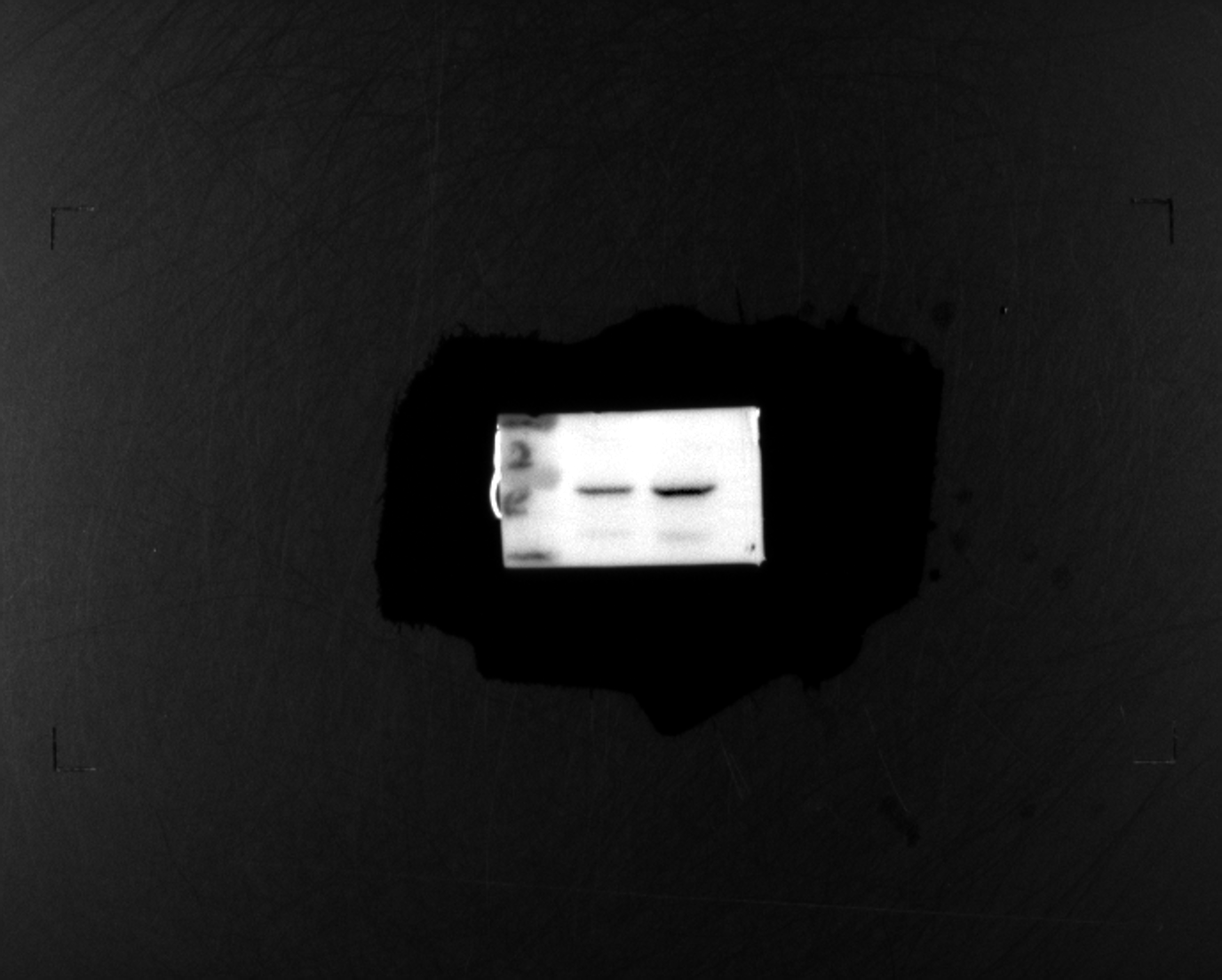

Supplement: Supplementary file 1 — Additional file 1. The original figures of the western blot. [file 12935_2023_2899_MOESM1_ESM.zip › Supplementary/Fig1.SETD8 Mia PaCa-2.Tif]

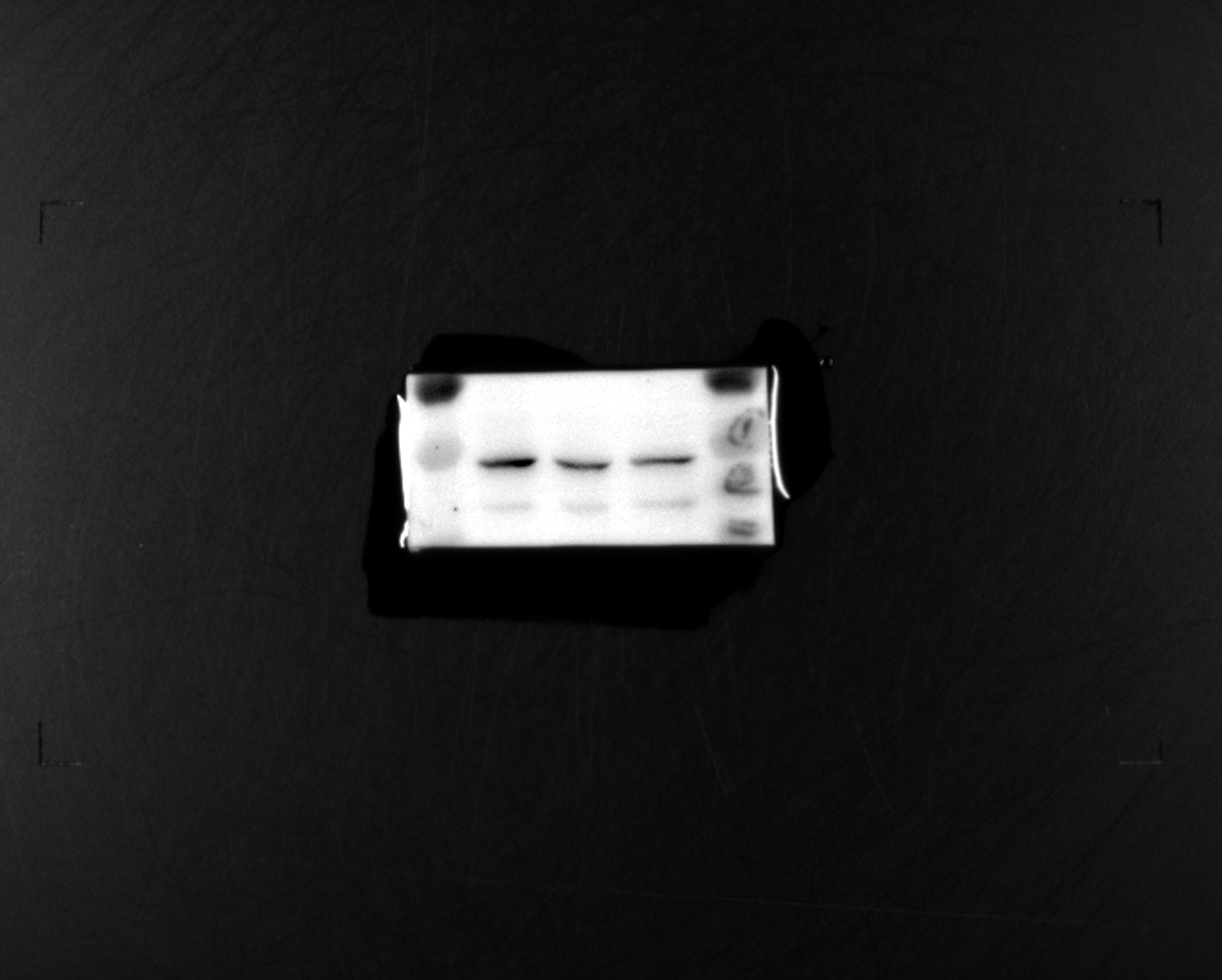

Supplement: Supplementary file 1 — Additional file 1. The original figures of the western blot. [file 12935_2023_2899_MOESM1_ESM.zip › Supplementary/Fig1.SETD8 SW1990.Tif]

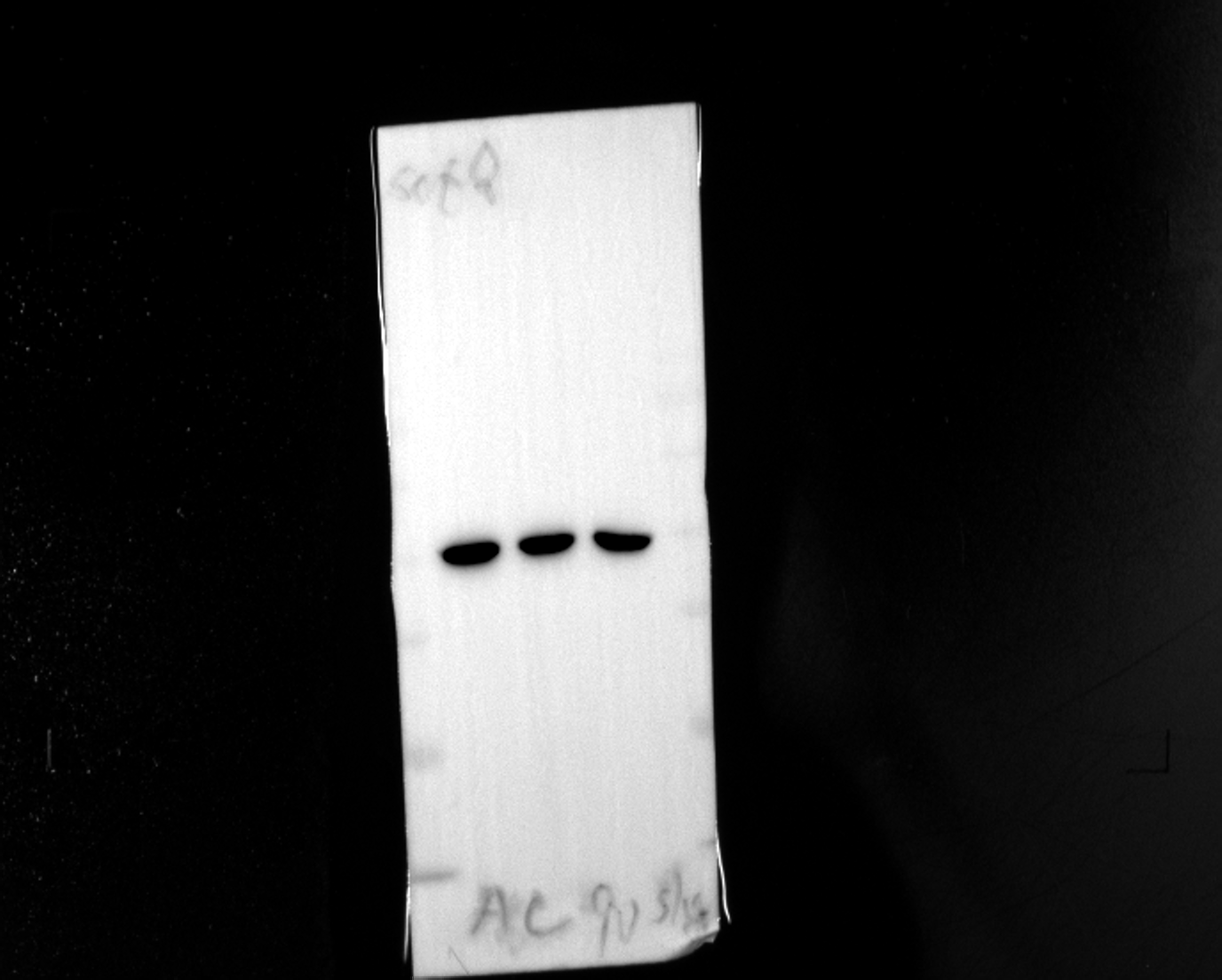

Supplement: Supplementary file 1 — Additional file 1. The original figures of the western blot. [file 12935_2023_2899_MOESM1_ESM.zip › Supplementary/Fig2 actin SW1990.Tif]

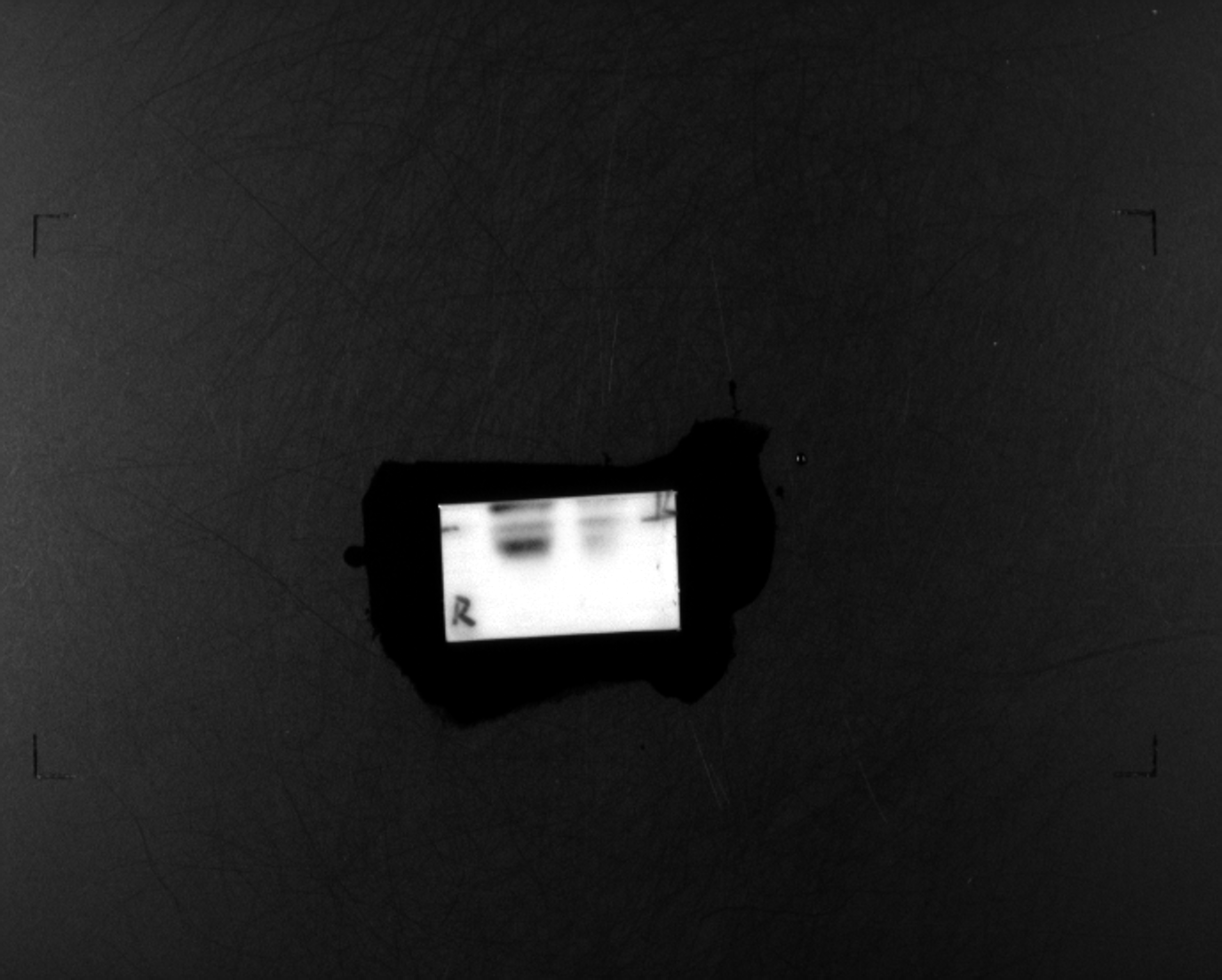

Supplement: Supplementary file 1 — Additional file 1. The original figures of the western blot. [file 12935_2023_2899_MOESM1_ESM.zip › Supplementary/Fig2.RRAD Mia PaCa-2.Tif]

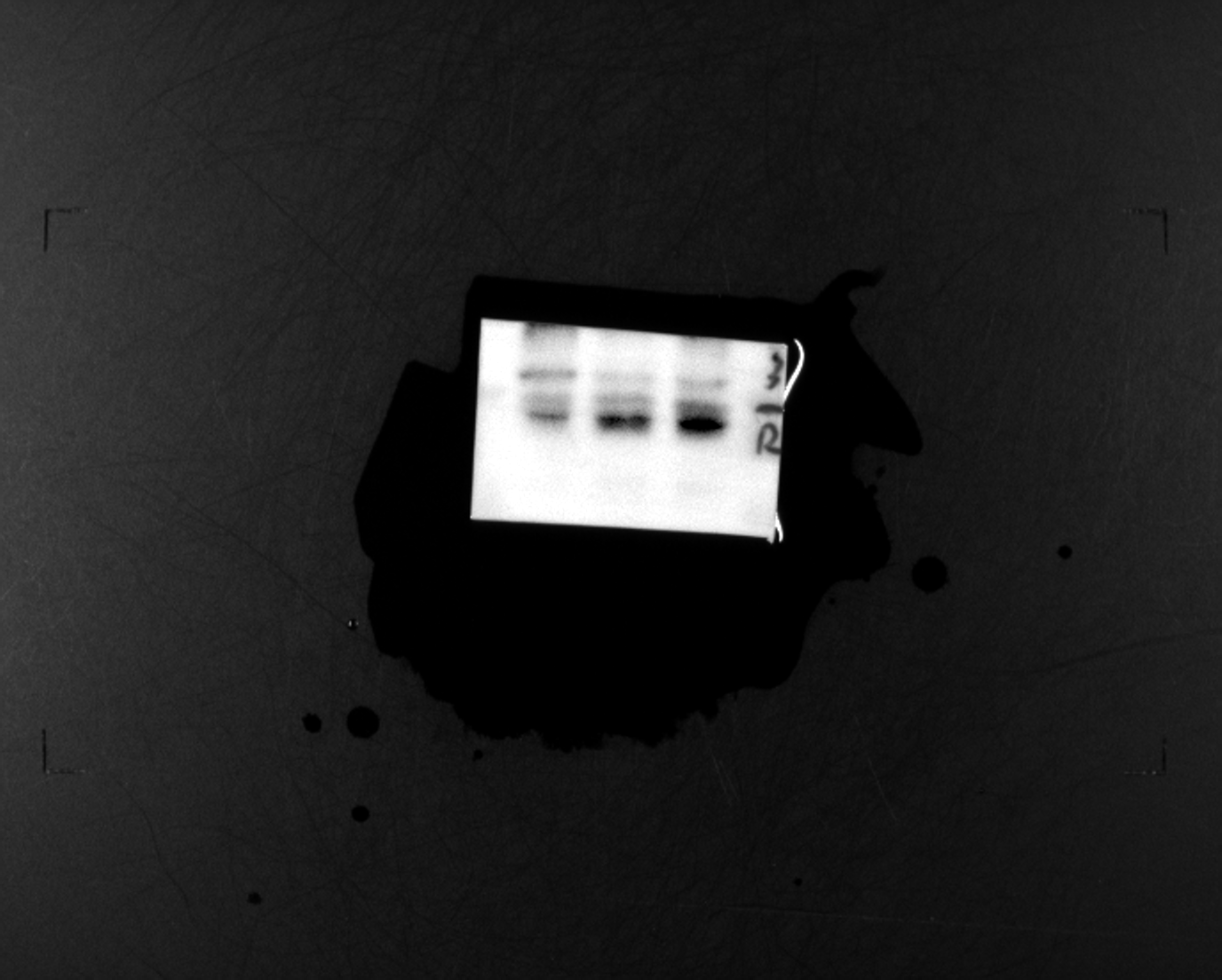

Supplement: Supplementary file 1 — Additional file 1. The original figures of the western blot. [file 12935_2023_2899_MOESM1_ESM.zip › Supplementary/Fig2.RRAD SW1990.Tif]

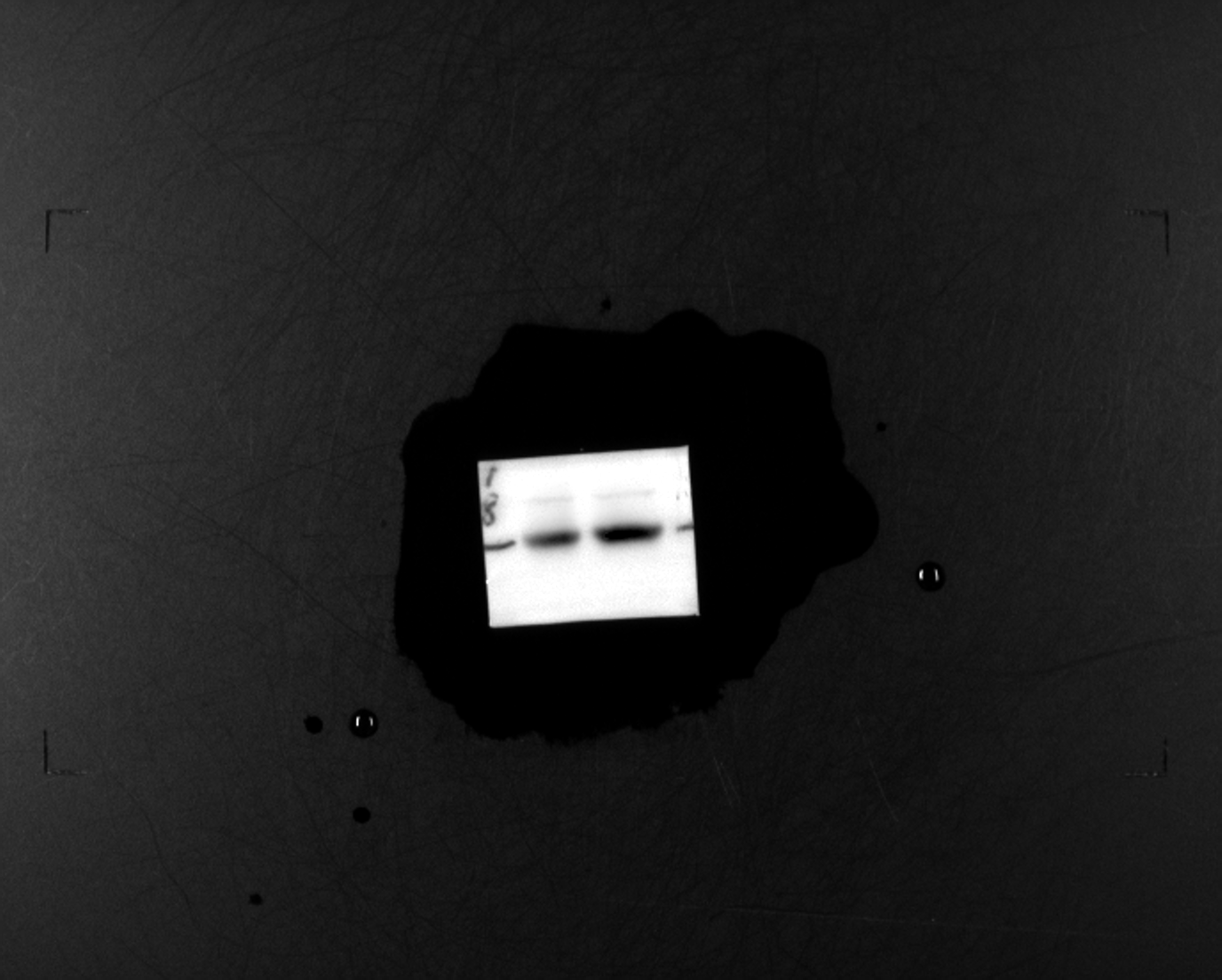

Supplement: Supplementary file 1 — Additional file 1. The original figures of the western blot. [file 12935_2023_2899_MOESM1_ESM.zip › Supplementary/Fig2.SETD8 Mia PaCa-2.Tif]

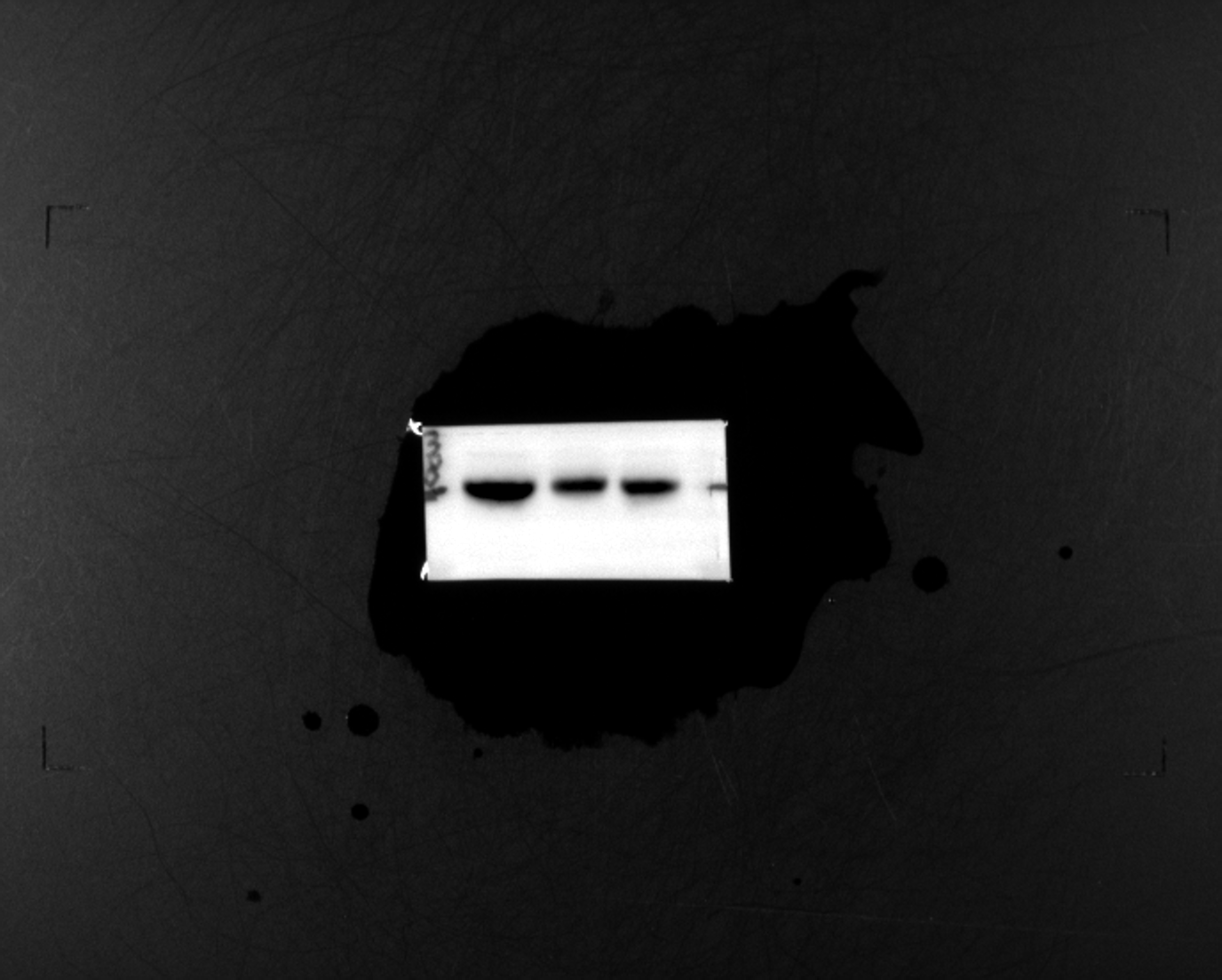

Supplement: Supplementary file 1 — Additional file 1. The original figures of the western blot. [file 12935_2023_2899_MOESM1_ESM.zip › Supplementary/Fig2.SETD8 SW1990.Tif]

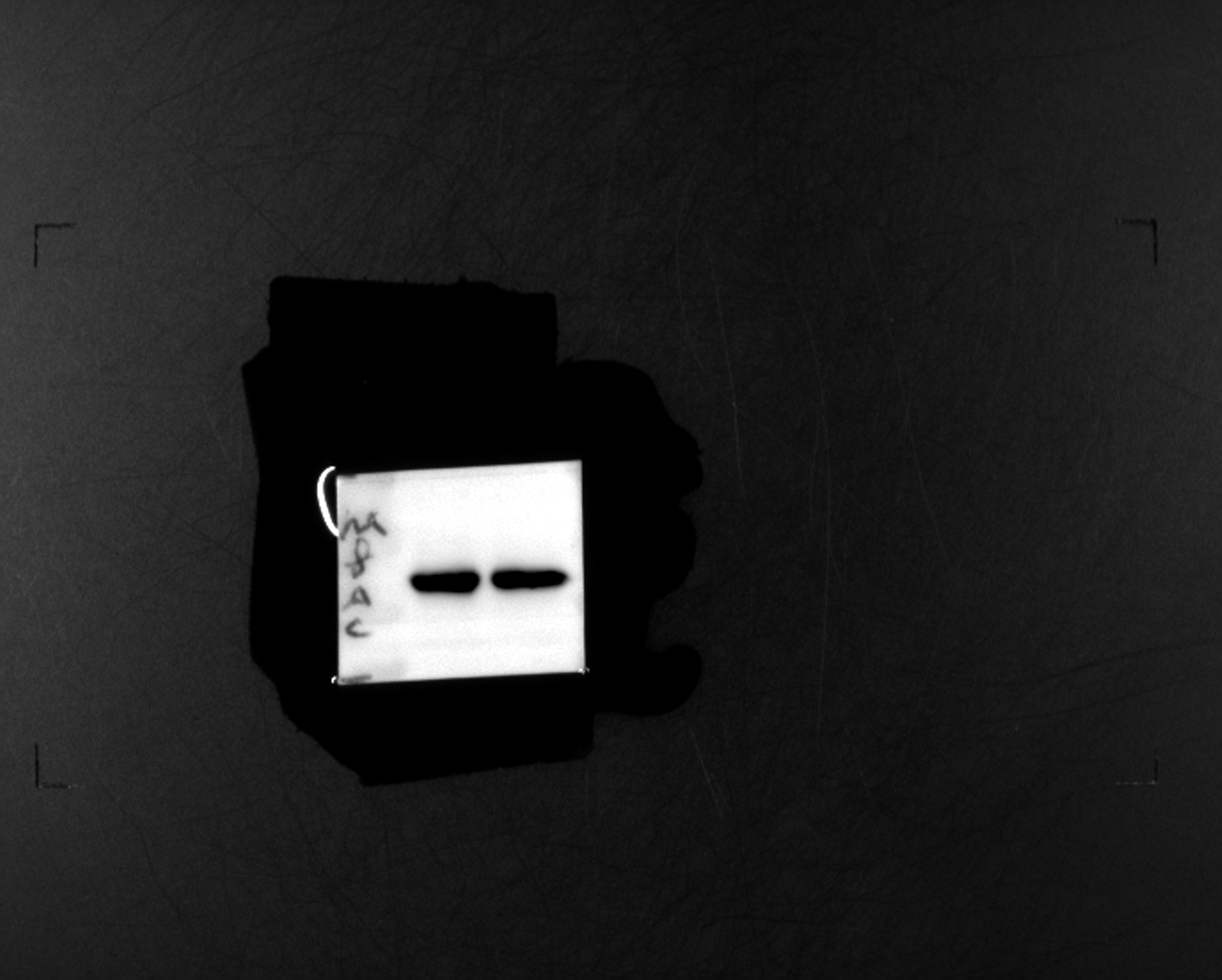

Supplement: Supplementary file 1 — Additional file 1. The original figures of the western blot. [file 12935_2023_2899_MOESM1_ESM.zip › Supplementary/Fig2actin Mia PaCa-2.Tif]

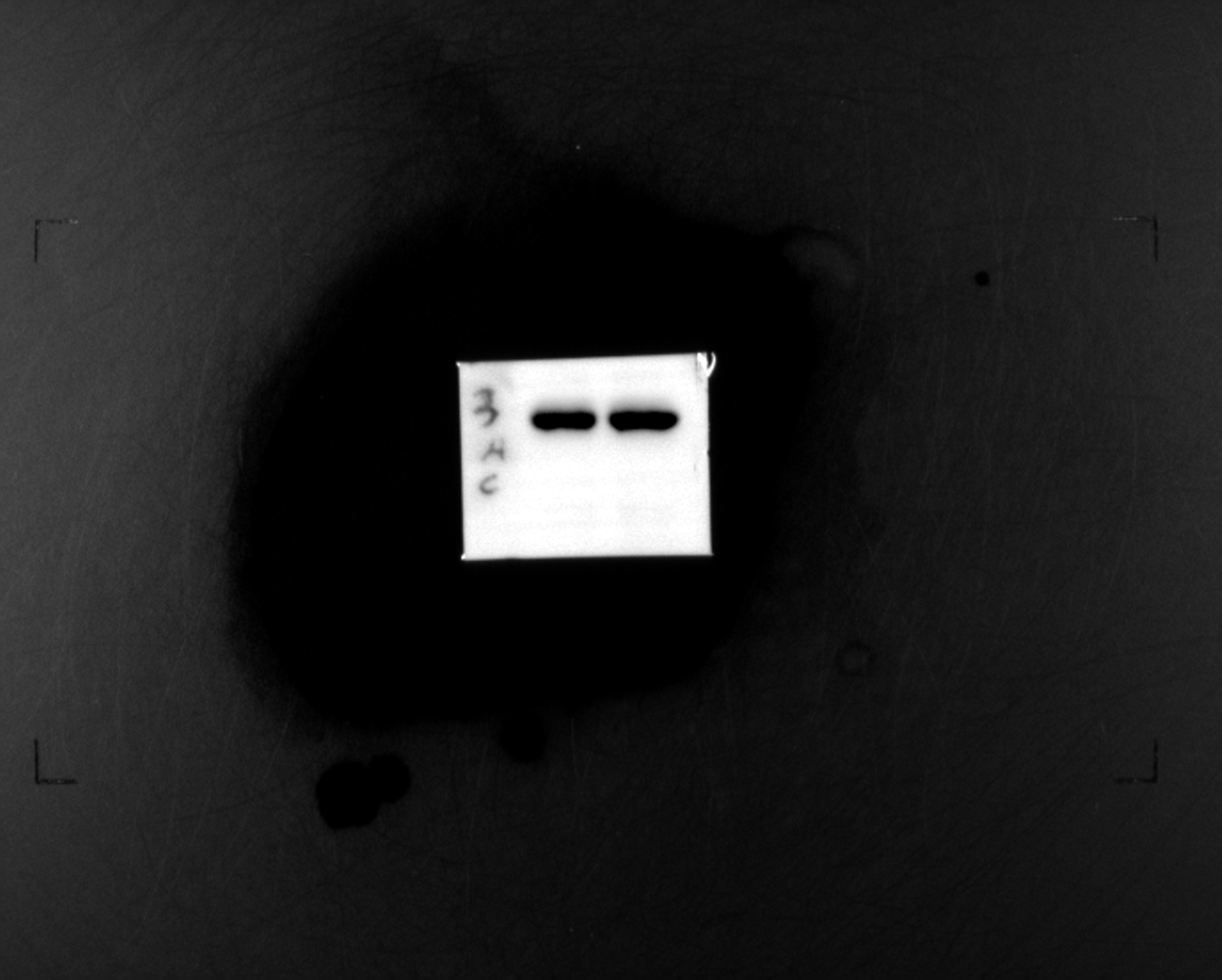

Supplement: Supplementary file 1 — Additional file 1. The original figures of the western blot. [file 12935_2023_2899_MOESM1_ESM.zip › Supplementary/Fig3 actin Mia PaCa-2.Tif]

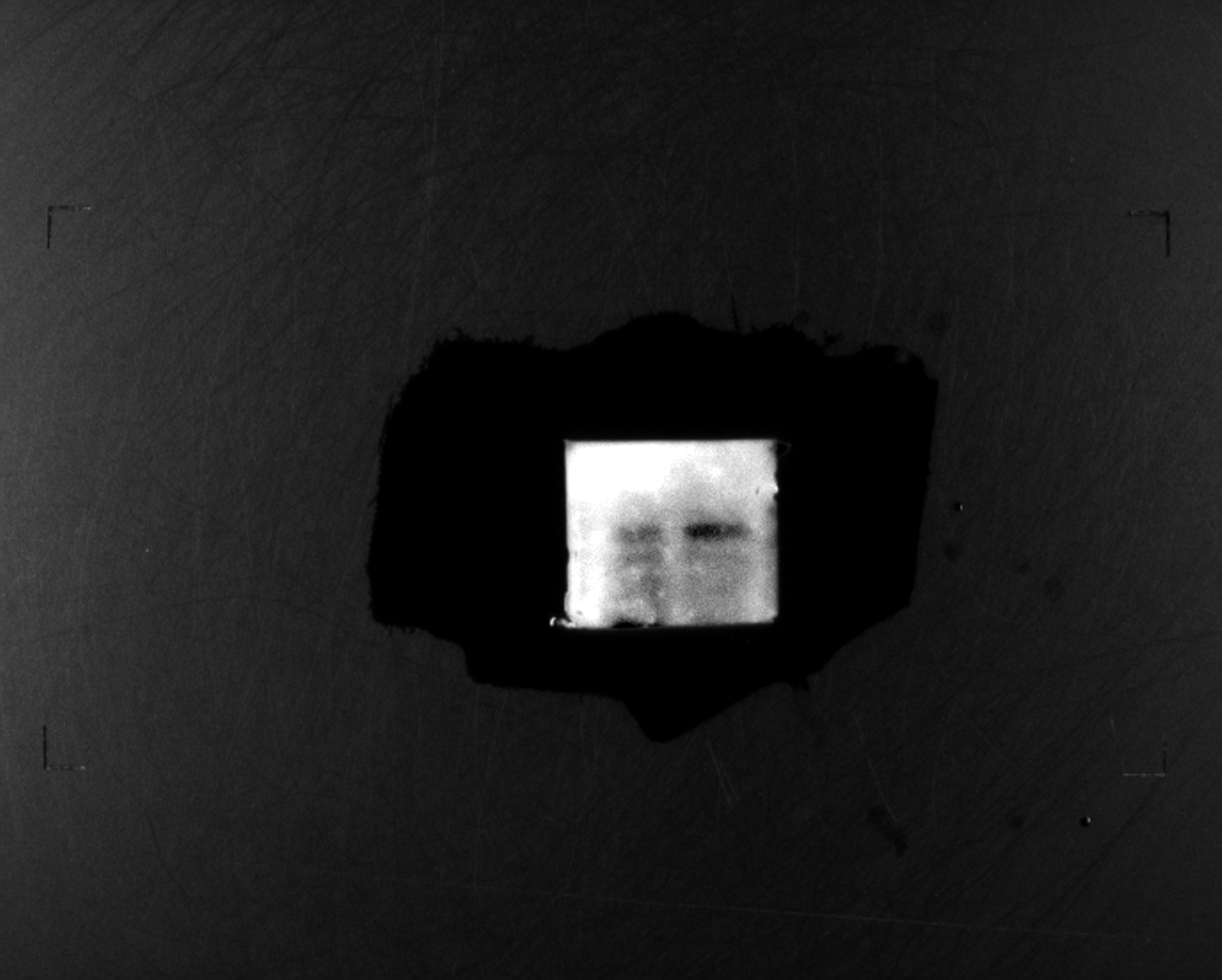

Supplement: Supplementary file 1 — Additional file 1. The original figures of the western blot. [file 12935_2023_2899_MOESM1_ESM.zip › Supplementary/Fig3.RRAD Mia PaCa-2.Tif]

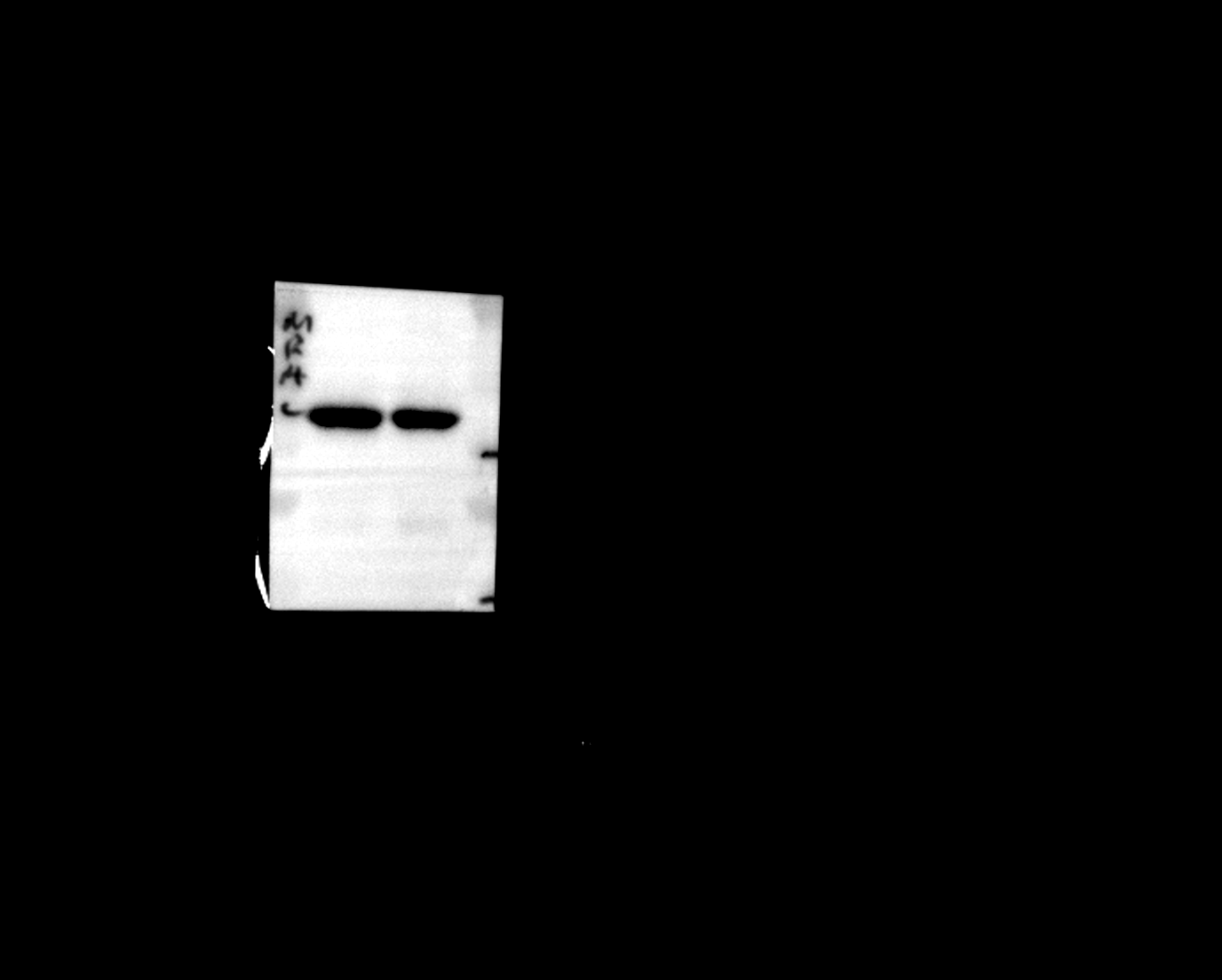

Supplement: Supplementary file 1 — Additional file 1. The original figures of the western blot. [file 12935_2023_2899_MOESM1_ESM.zip › Supplementary/Fig4. actin Mia PaCa-2.Tif]

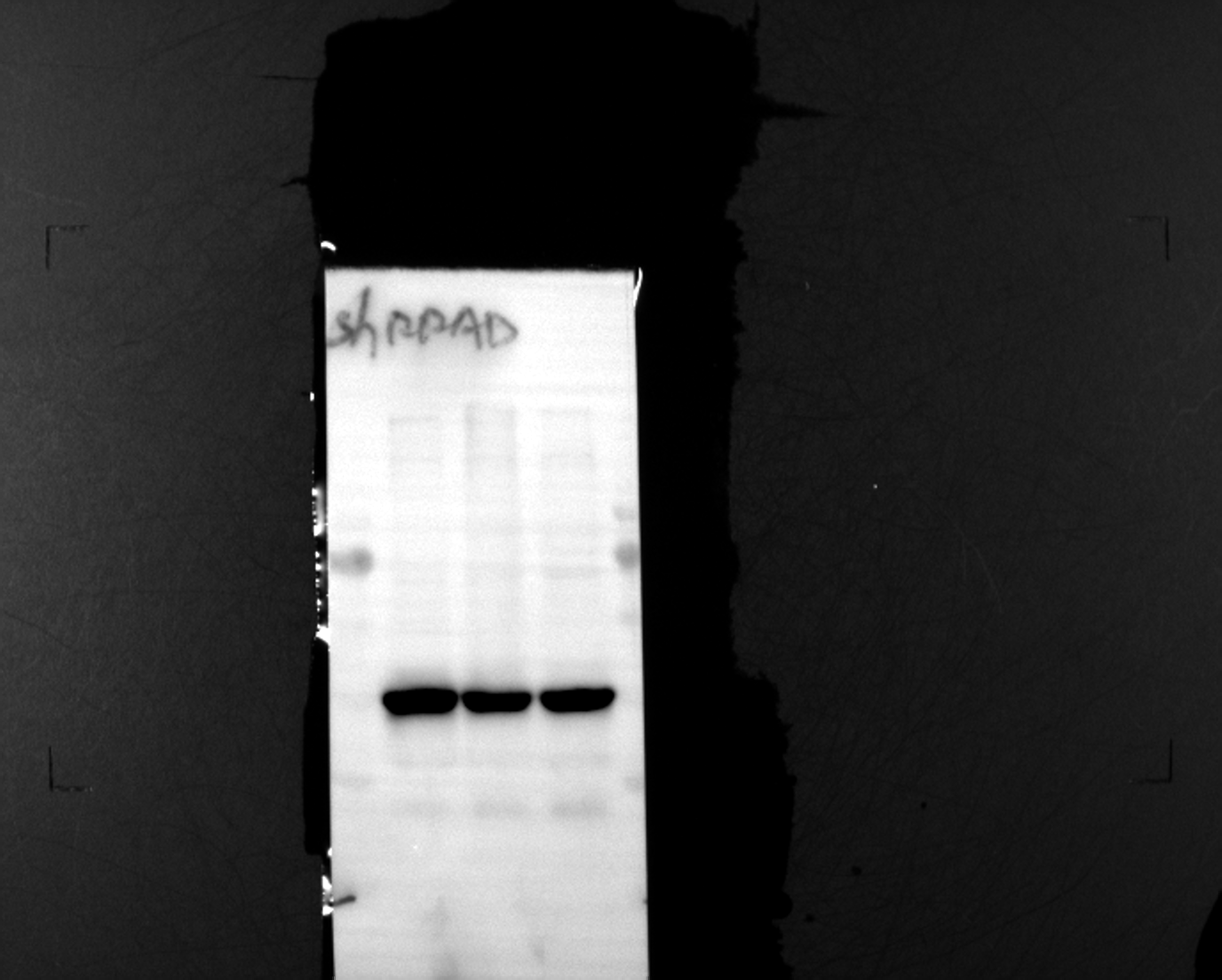

Supplement: Supplementary file 1 — Additional file 1. The original figures of the western blot. [file 12935_2023_2899_MOESM1_ESM.zip › Supplementary/Fig4. actin SW1990.Tif]

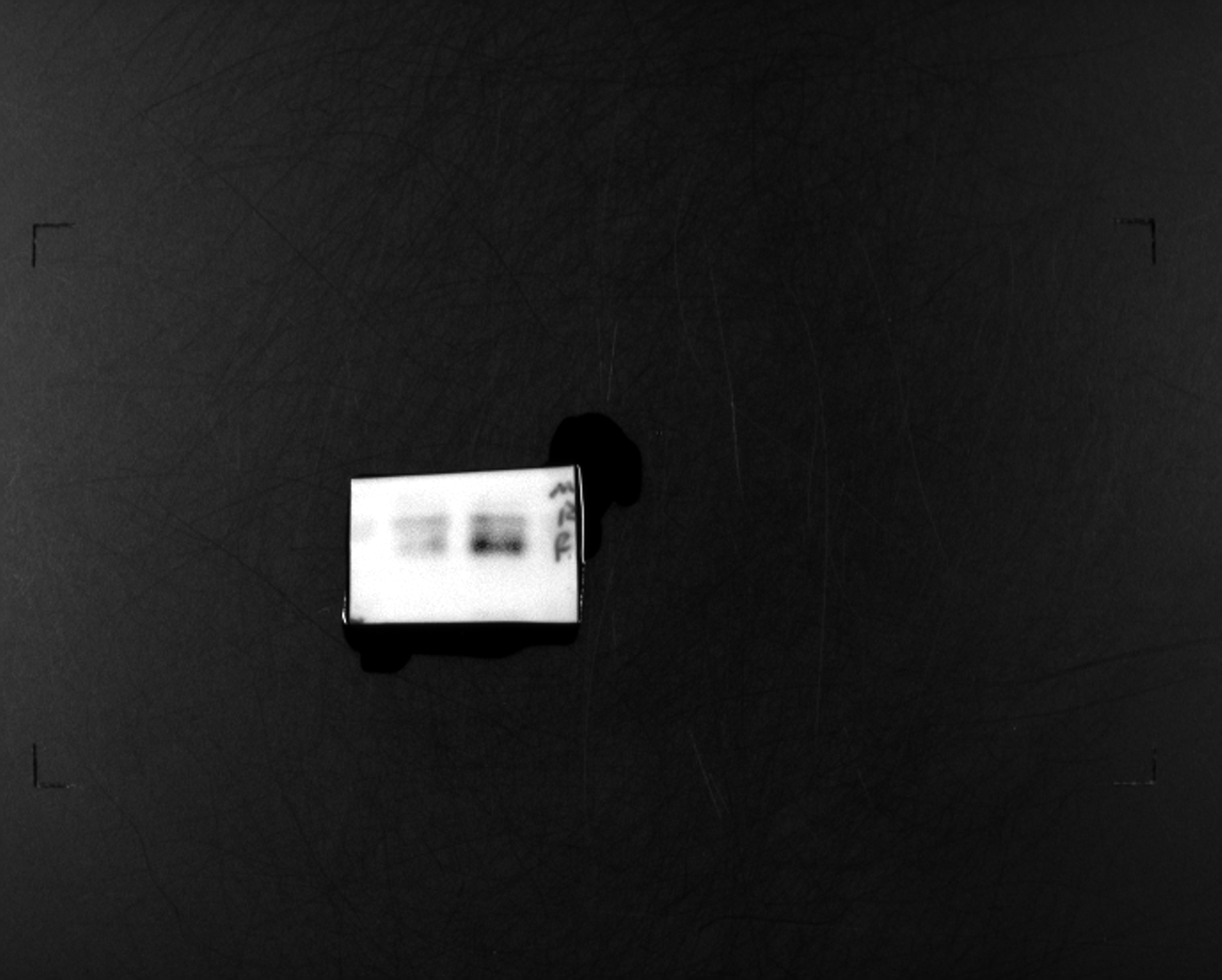

Supplement: Supplementary file 1 — Additional file 1. The original figures of the western blot. [file 12935_2023_2899_MOESM1_ESM.zip › Supplementary/Fig4.RRAD Mia PaCa-2.Tif]

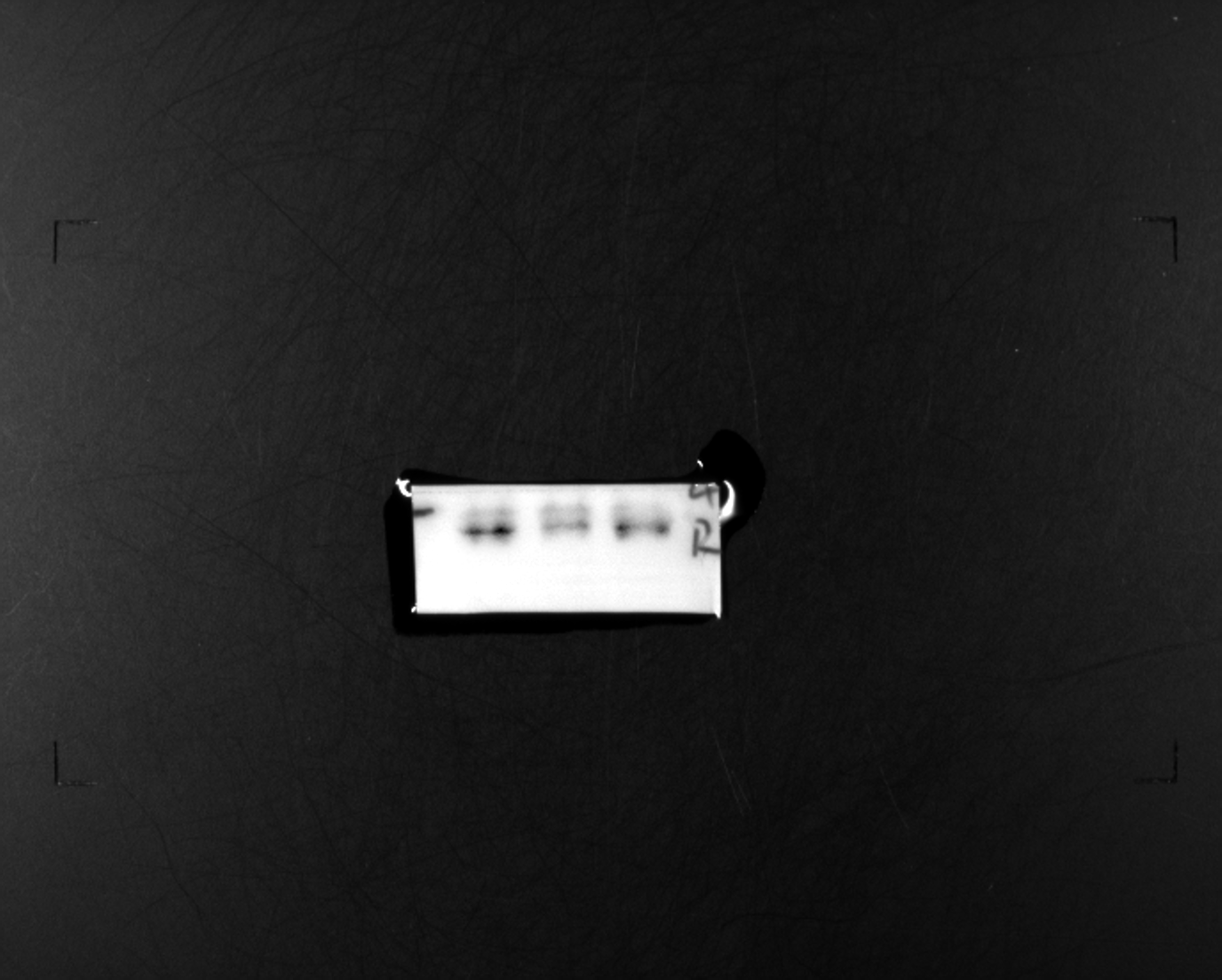

Supplement: Supplementary file 1 — Additional file 1. The original figures of the western blot. [file 12935_2023_2899_MOESM1_ESM.zip › Supplementary/Fig4.RRAD SW1990.Tif]

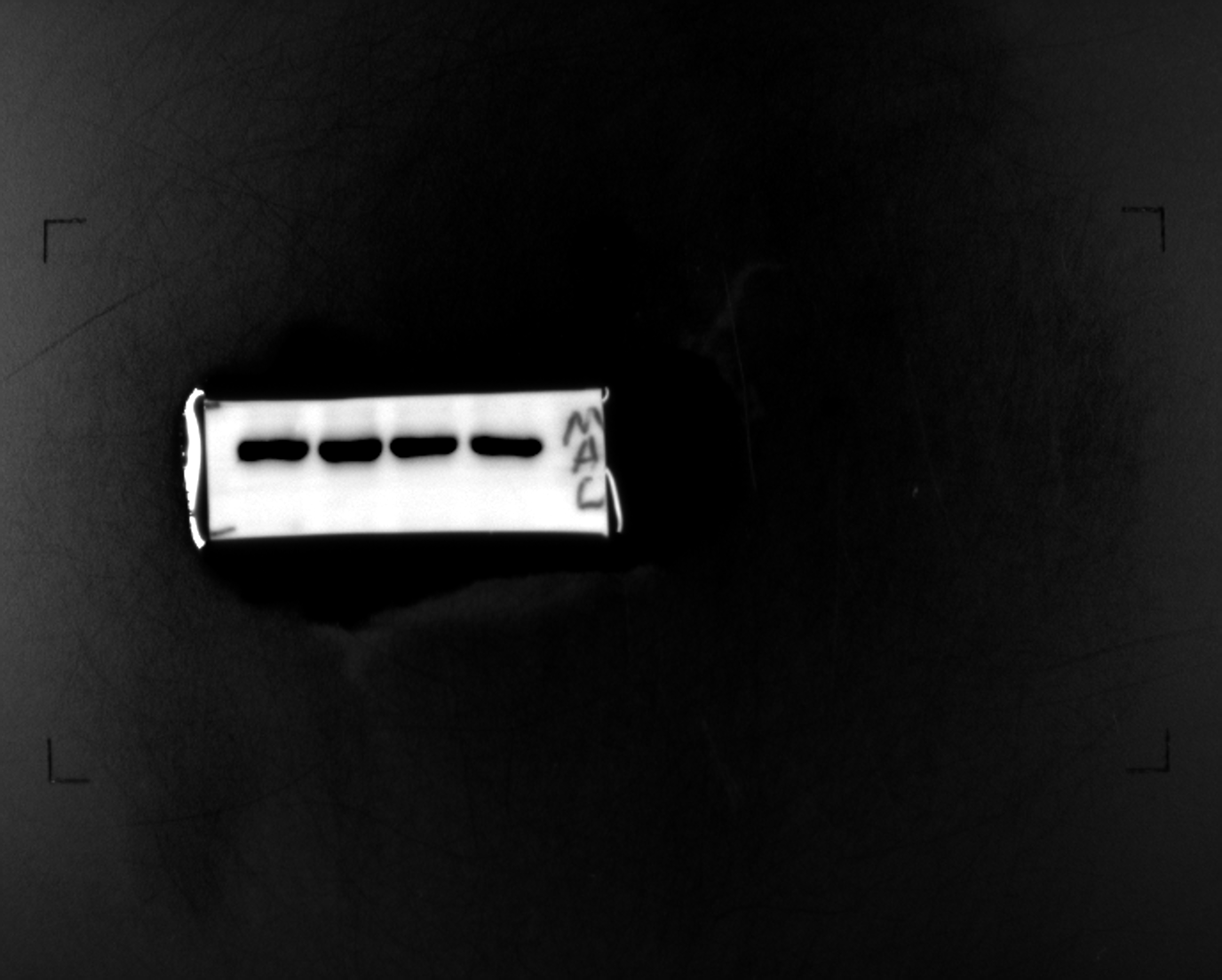

Supplement: Supplementary file 1 — Additional file 1. The original figures of the western blot. [file 12935_2023_2899_MOESM1_ESM.zip › Supplementary/Fig5 actin Mia PaCa-2.Tif]

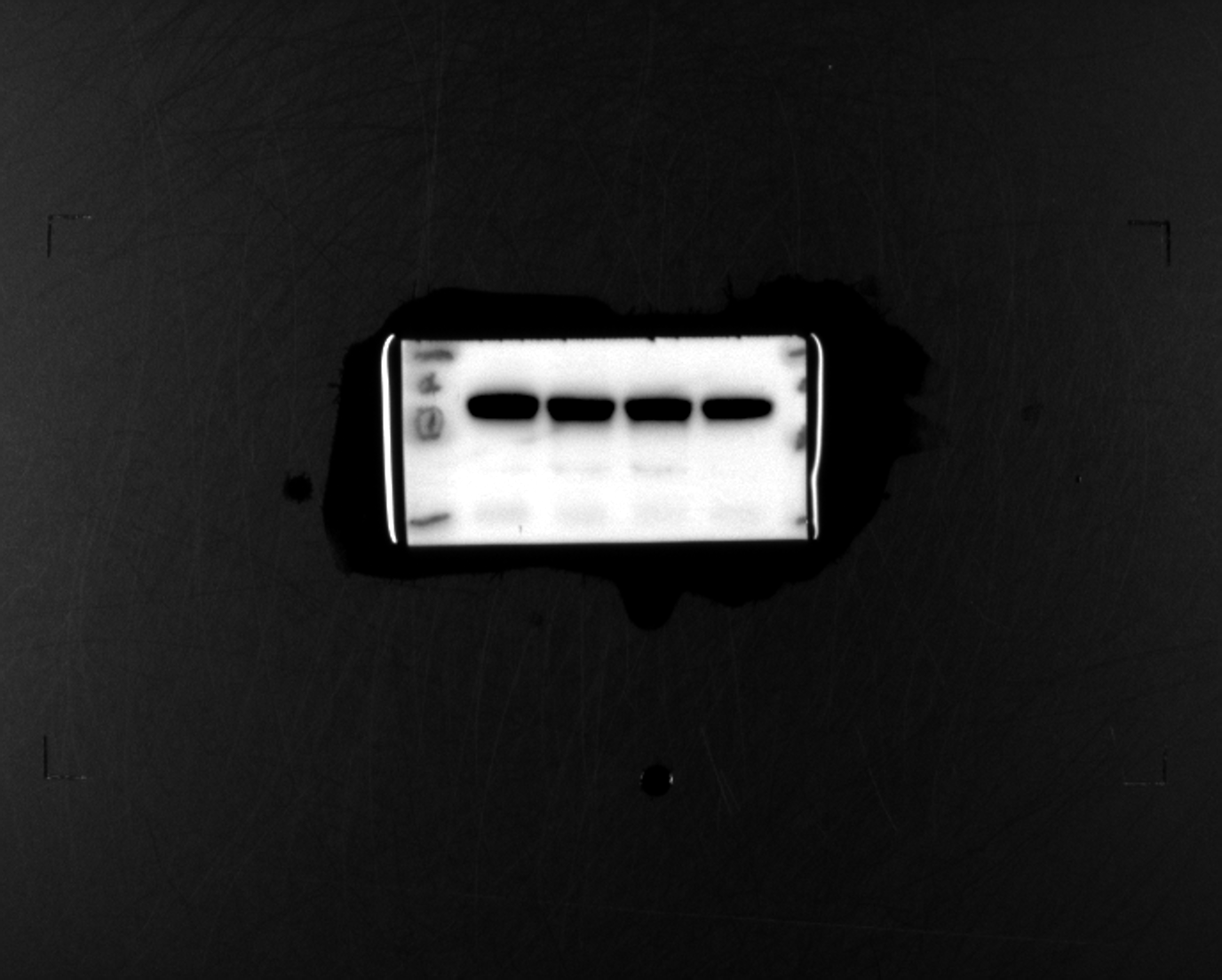

Supplement: Supplementary file 1 — Additional file 1. The original figures of the western blot. [file 12935_2023_2899_MOESM1_ESM.zip › Supplementary/Fig5 actin SW1990.Tif]

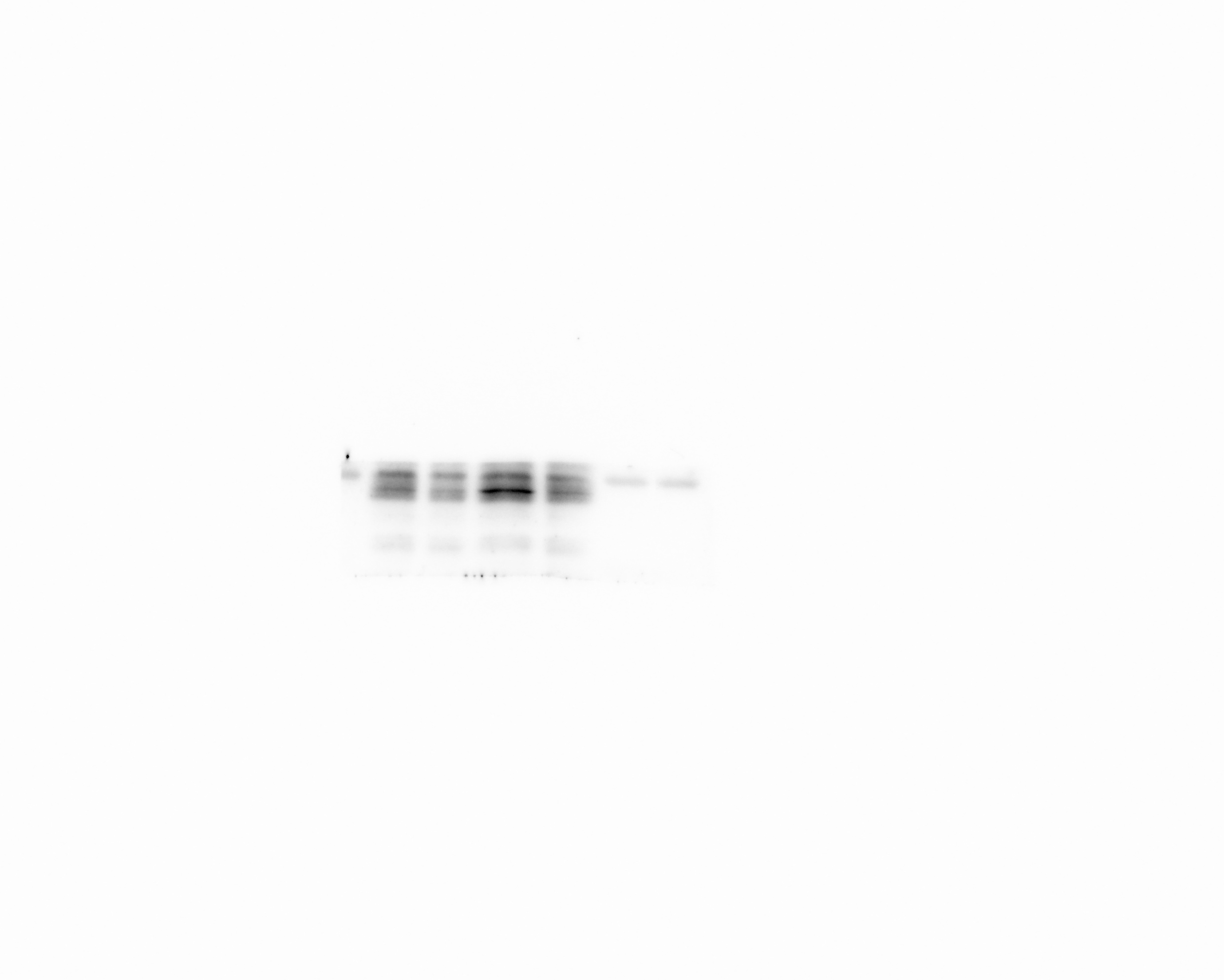

Supplement: Supplementary file 1 — Additional file 1. The original figures of the western blot. [file 12935_2023_2899_MOESM1_ESM.zip › Supplementary/Fig5.RRAD Mia PaCa-2.jpg]

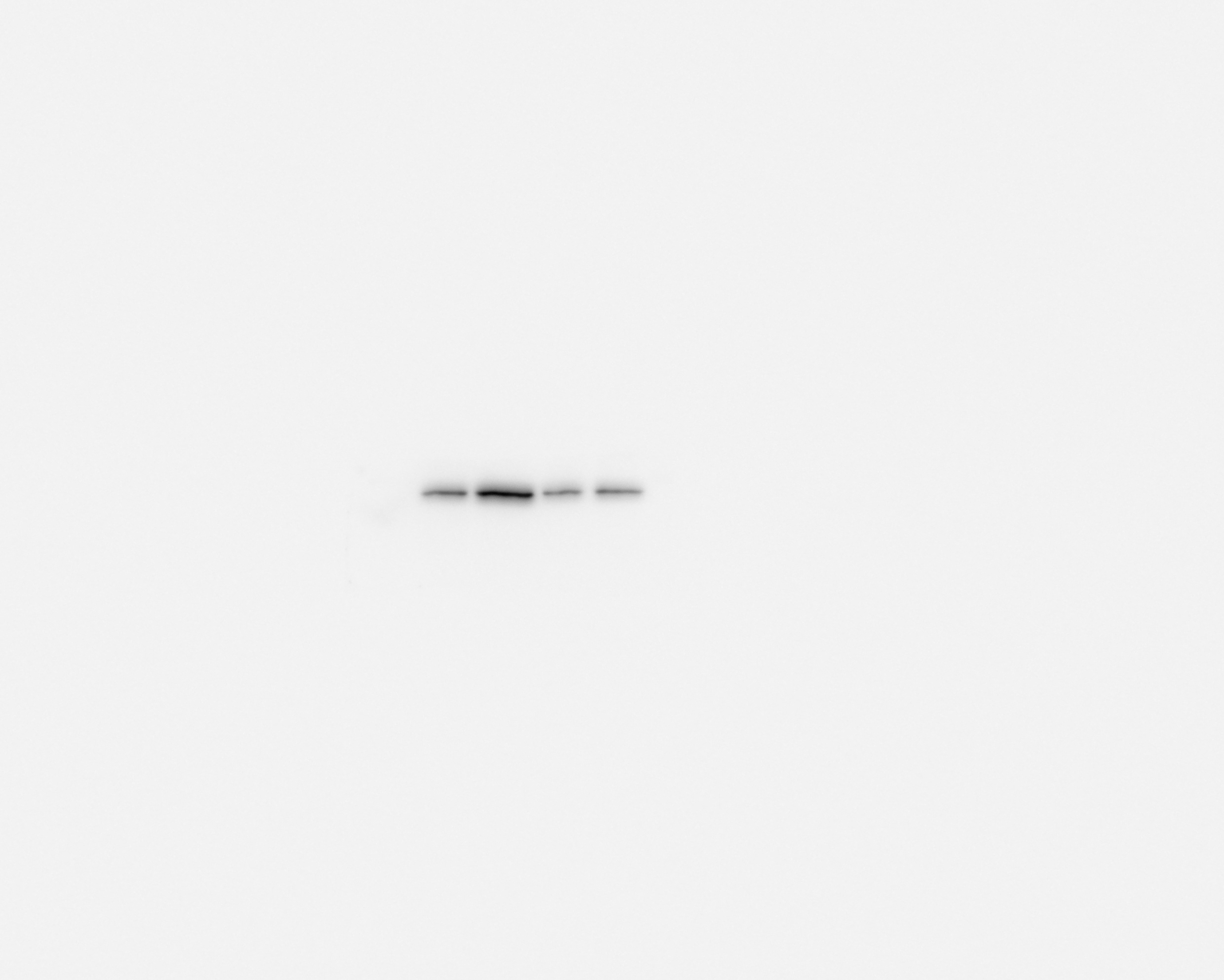

Supplement: Supplementary file 1 — Additional file 1. The original figures of the western blot. [file 12935_2023_2899_MOESM1_ESM.zip › Supplementary/Fig5.RRADSW1990.jpg]

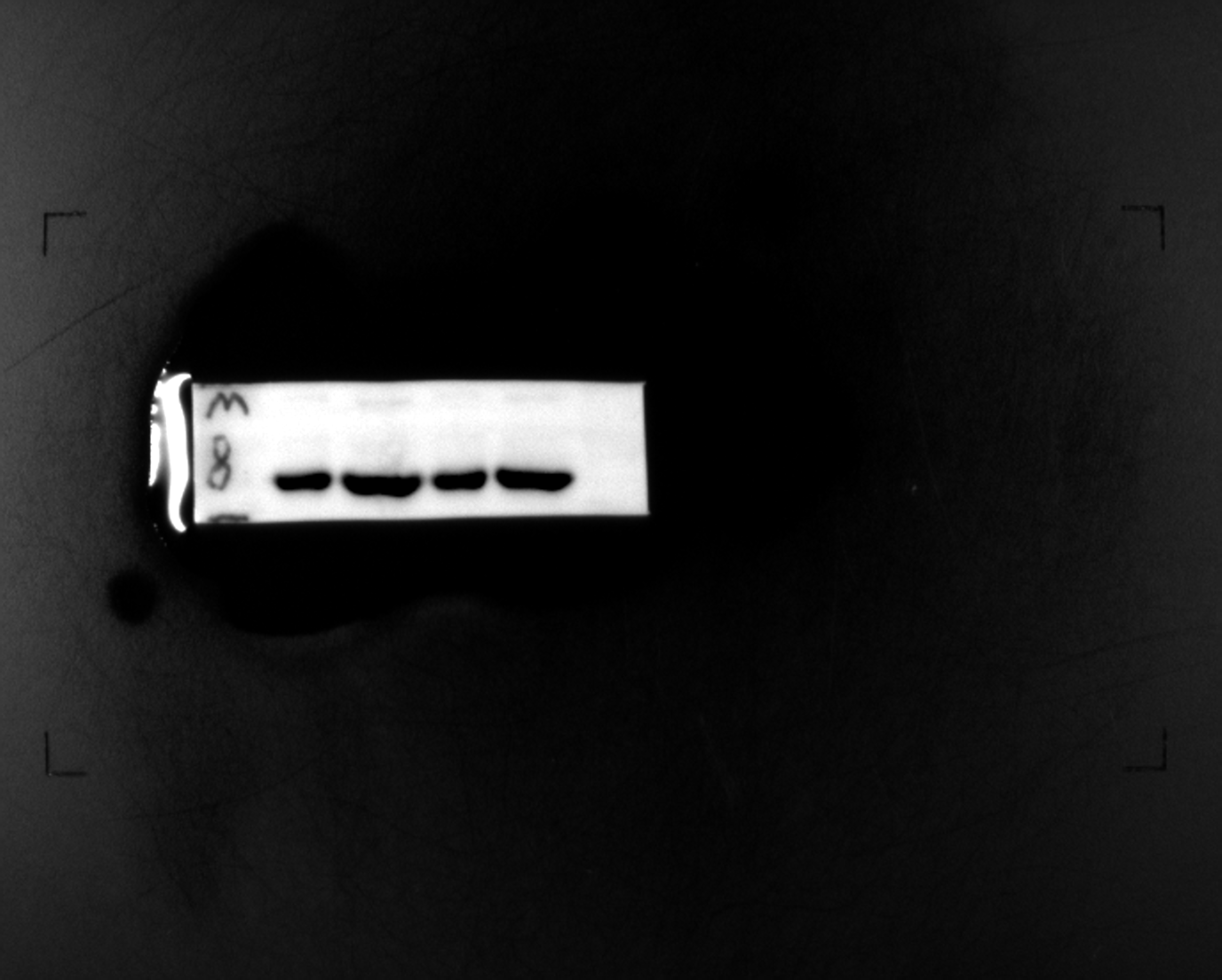

Supplement: Supplementary file 1 — Additional file 1. The original figures of the western blot. [file 12935_2023_2899_MOESM1_ESM.zip › Supplementary/Fig5.SETD8 Mia PaCa-2.Tif]

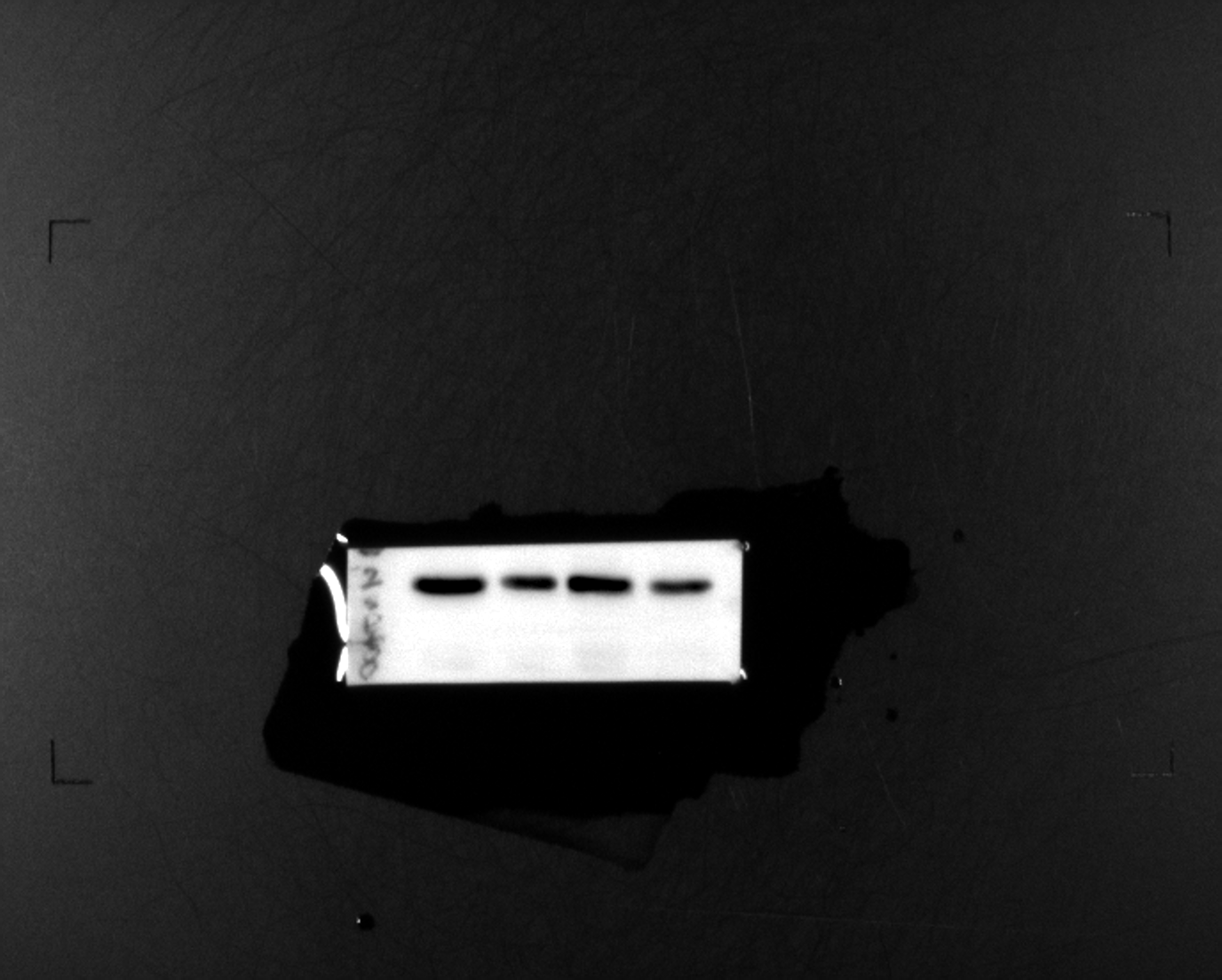

Supplement: Supplementary file 1 — Additional file 1. The original figures of the western blot. [file 12935_2023_2899_MOESM1_ESM.zip › Supplementary/Fig5.SETD8SW1990.Tif]
